# Transformation of SOX9^+^ cells by* Pten* deletion synergizes with steatotic liver injury to drive development of hepatocellular and cholangiocarcinoma

**DOI:** 10.1038/s41598-021-90958-1

**Published:** 2021-06-03

**Authors:** Jingyu Chen, Anketse Debebe, Ni Zeng, Janel Kopp, Lina He, Maike Sander, Bangyan L. Stiles

**Affiliations:** 1 Pharmacology and Pharmaceutical Sciences, School of Pharmacy, University of Southern California, Los Angeles, CA 90033 USA; 2 Department of Cellular and Physiological Sciences, University of British Columbia, Vancouver, BC V6T 1Z3 Canada; 3 Department of Pediatrics and Cellar and Molecular Medicine, UCSD, La Jolla, CA 92093 USA; 4 Department of Pathology, Keck School of Medicine, University of Southern California, Los Angeles, CA 90033 USA

**Keywords:** Cancer, Cell biology, Molecular biology, Gastroenterology

## Abstract

SOX9 (Sex-determining region Y Box 9) is a well-characterized transcription factor that is a marker for progenitor cells in various tissues. In the liver, cells delineated by SOX9 are responsible for regenerating liver parenchyma when cell proliferation is impaired following chronic injury. However, whether these SOX9^+^ cells play a role in liver carcinogenesis has not been fully understood, although high SOX9 expression has been linked to poor survival outcome in liver cancer patients. To address this question, we developed a liver cancer mouse model (*Pten*^*loxP/loxP*^; *Sox9-Cre*^*ERT*+^*; R26R*^*YFP*^) where tumor suppressor *Pten* (phosphatase and tensin homolog deleted on chromosome ten) is deleted in SOX9^+^ cells following tamoxifen injection. In this paper, we employ lineage-tracing to demonstrate the tumorigenicity potential of the *Pten*^-^, SOX9^+^ cells. We show that these cells are capable of giving rise to mixed-lineage tumors that manifest features of both hepatocellular carcinoma (HCC) and intrahepatic cholangiocarcinoma (CCA). Our results suggest that PTEN loss induces the transformation of SOX9^+^ cells. We further show that to activate these transformed SOX9^+^ cells, the presence of liver injury is crucial. Liver injury, induced by hepatotoxin 3,5-diethoxycarbonyl-1,4-dihydrocollidine (DDC) or high-fat diet (HFD), substantially increases tumor incidence and accelerates liver carcinogenesis from SOX9^+^ cells in *Pten* null mice but not in control mice. We further examine the mechanisms underlying tumor formation in this model to show that concurrent with the induction of niche signal (i.e., Wnt signaling), liver injury significantly stimulates the expansion of tumor-initiating cells (TICs). Together, these data show that (1) SOX9^+^ cells have the potential to become TICs following the primary transformation (i.e. *Pten* deletion) and that (2) liver injury is necessary for promoting the activation and proliferation of transformed SOX9^+^ cells, resulting in the genesis of mixed-lineage liver tumors.

## Introduction

Liver cancer is the sixth most common cancer worldwide and the most rapidly growing malignancy in the US^1,2^. Primary liver cancer is a heterogeneous group of malignancies that include both hepatocellular carcinoma (HCC) and intrahepatic cholangiocarcinoma (CCA) as the two most common subtypes. Using genomic, transcriptomic, and metabolic profiling, a recent study revealed that there is a continuum of overlapping neoplasms among all subtypes of liver cancer, with some predominately exhibiting HCC or CCA while others manifest a mixed phenotype of both to varying degrees^3,4^. Tumors exhibiting combined HCC and CCA phenotypes display more aggressive behavior and are more likely to relapse after resection treatment. SOX9 (sex-determining region Y Box 9) is one of several related high mobility group box transcriptional factors that play essential roles in the embryonic development of many tissues and organs including the liver^5^. In adult livers, SOX9 expression is primarily observed in the cholangiocytes that line the bile ducts. Recent studies also observed low level expression of SOX9 in hepatocytes surrounding the ductular structures and found that SOX9^+^ cells can repair liver injury induced damages^6,7^. In liver cancer, high SOX9 expression correlates with advanced tumor stage, higher tumor grade, poorer recurrence-free survival, and poorer overall survival^8,9^. In HCC cell lines ectopically expressing Sox9-eGFP (SOX9 promoter driven enhanced green fluorescent protein), FACS-isolated SOX9^+^ cells are found to be the tumor forming cells^10,11^.

In this paper, we demonstrate the tumorigenicity of these SOX9^+^ cells by targeted deletion of the tumor suppressor *Pten* (phosphatase and tensin homologue deleted on chromosome 10). PTEN is a negative regulator of the phosphatidylinositol 3-kinase (PI3K)/AKT pathway. *Pten* is the 2^nd^ most deleted tumor suppressor gene in human cancers. Expression of PTEN is lost or downregulated in approximately 50% of HCC^12,13^ and 70% of CCA^14^, respectively. In tumors where PTEN remains positive, it is reported to be inactivated via phosphorylation (S380, T382/383) in 89% of tumor samples^14^. Previous studies from our group showed that deletion of *Pten* in albumin expressing liver cells (*Pten*^*loxP/loxP*^*; Alb-Cre*^+^, Alb-Pten) resulted in large scale hepatocyte cell death followed by tumor formation and that inhibiting liver injury blocks tumor development in these mice^15,16^. To explore the relationship between liver injury and tumor formation; and particularly the cell types that undergo each of these two processes (tumor formation vs. liver injury), we targeted the deletion of *Pten* to the Sox9^+^ cells in the adult liver (Sox9-Pten*, Pten*^*loxP/loxP*^*; Sox9-Cre*^*ERT*+^*; R26R*^*YFP*^ + tamoxifen). We explored if SOX9^+^ cells are the tumor forming cells activated upon PTEN loss; and whether liver injury promotes the formation of tumors from these *Pten*-deleted SOX9^+^ cells. Our results show that deletion of *Pten* in the SOX9^+^ cells can lead to mixed-lineage liver tumor formation. We further establish that liver injury propagates the genotoxic events caused by PTEN loss and is essential for tumors to develop from the *Pten* deletion-transformed SOX9^+^ cells. With the presence of steatosis or chemical induced injury, SOX9^+^ cells accumulate in the liver, resulting in a remarkably increased incidence and early onset of liver tumors, all originating from transformed SOX9^+^ cells.

## Results

### ***Pten*** loss induces the transformation of SOX9^+^ cells

To accomplish the deletion of *Pten*, tamoxifen was subcutaneously injected in three doses separated by two-day intervals at 1 month of age to the *Pten*^*loxP/loxP*^*;Sox9-Cre*^*ERT*+^*; R26R*^*YFP*^ mice (supplemental Fig. 1A). Control mice are either the same genotype mice injected with corn oil (vehicle), *Pten *^*loxP/loxP*^*; Sox9-Cre*^*-*^ mice with no treatment; or *Pten*^+*/*+^*; Sox9-Cre*^*ERT*+^ mice treated with either corn oil (CO) or tamoxifen. Consistent with the endogenous expression pattern of SOX9^17^, we observed YFP expression in multiple tissues, including testes, pancreas, stomach, eye, and brain, after tamoxifen injection (data not shown). While the body weight of Sox9-Pten mice was moderately lower than the controls 12 months after injection (supplemental Fig. 1B), no other gross morphological changes were observed with the exception of excessive eye discharge from the Sox9-Cre mice regardless of tamoxifen injection.

**Figure 1:**
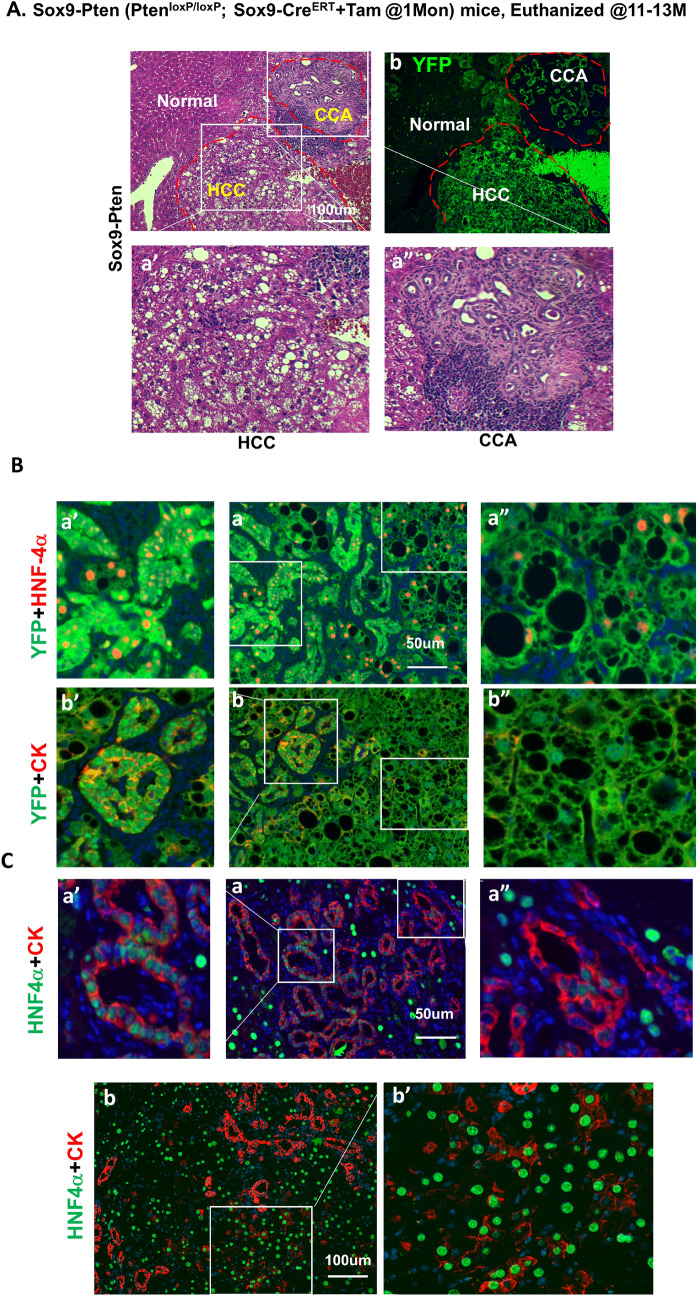
HCC and CCA developed in the aged Sox9-Pten mice. (**A**) Phenotype of tumors developed in the 11–13 months old Sox9-Pten mice. H&E (a, a′ and a″) and immunofluorescent staining of YFP (b) on serial sections of the tumors developed in Sox9-Pten mice at 11–13 months old. Panels a′ and a″ are amplified views of the boxed areas in panel a. H&E staining shows tumors that morphologically resembles HCC (a′) and CCA (a″). (**B**) Immunofluorescent staining showing presence of both HCC composed of hepatocytes and CCA composed of cholangiocytes in the YFP positive tumors. Antibodies used are: HNF4α (red) for hepatocytes (a, a′ and a″), CK (red) for cholangiocytes (b, b′ and b″) and YFP. YFP positive cells indicate SOX9^+^ cells and their progenies that were genetically manipulated to lack PTEN. a′ and b′, enlarged view of morphologically ductal structures within the tumor. a″ and b″, enlarged view of morphologically hepatocyte structures within the tumor. (**C**) Co-immunofluorescent staining for HNF4α (green) and CK (red) shows tumor cells that express both markers in different areas of the tumor. Panels a, a′ and a″ depict morphological ductal structures that express hepatocytes marker HNF4α (green) in addition to CK (red) which is a marker for cholangiocytes; Panels b and b′ depict morphological hepatic structures that express cholangiocyte marker CK (red) in addition to HNF4α (green) which is a marker for hepatocytes. Blue, DAPI to mark nuclei.

In the liver, expression of YFP was observed at the periportal area 3 days after the last dose of tamoxifen injection (supplemental Fig. 1C), consistent with the expression pattern of SOX9 in the liver^17^. At this time, no obvious difference was observed morphologically, histologically or phenotypically between the Sox9-Pten and control livers. We then analyzed the morphology of the livers in these mice at different ages. In mice at 6 and 8.5–9.5 months of age, mild to moderate reactive duct/oval cell activation phenotype was observed in the Sox9-Pten livers (supplemental Fig. 2). Approximately 50–60% of the mice developed von Meyenburg complex (VMC) at these ages, whereas the control mice had a healthy liver morphology. In the cohort of mice at 11–13 months old, all but one mouse analyzed developed severe oval cell activation and ductal reaction in the periportal area of the liver. In addition, over 80% of the Sox9-Pten mice developed VMC condition and 63.6% (14 out of 22) of them had liver tumors at this age, in contrast with 0% in control mice (0 out of 13). Extensive fibrosis is also observed in the Sox9-Pten mice 6 months old and older, evidenced by the deposition of collagen fiber stained with Sirius red (supplemental Fig. 3). The expression of collagen and smooth muscle α actin is also significantly upregulated. Table 1 quantifies the occurrences the various anomalous phenotypes at different ages. Supplemental Fig. 2 depicts example images of the various phenotypes for the different age groups with controls for comparison.

**Figure 2:**
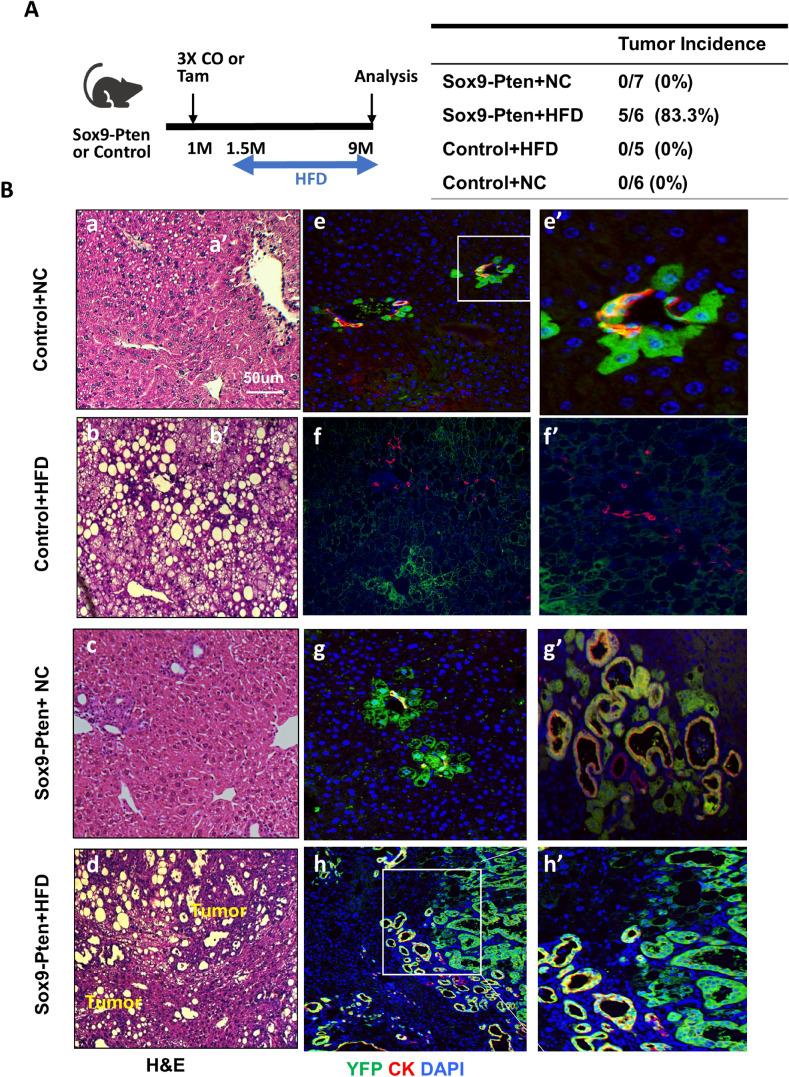
High fat diet promotes liver cancer development in Sox9-Pten mice. (**A**) Left, tamoxifen injection and diet protocol. Diet is initiated at 1.5 month and tissues collected at 9 months of age. Right, quantitative analysis of tumor development in control and Sox-Pten mice on normal chow (NC) and high fat diet (HFD). (**B**) Representative microscopic images of the livers from Sox9-Pten and control mice on HFD. Panels a-d, H&E to indicate morphology of the liver sections. Liver tumors were only observed in HFD fed Sox9-Pten mice. Panels e–h and e′-h′ are stained with YFP (green) + CK (red). e′ and h′ are amplified views of the boxed areas of e and h respectively. f′ is amplified views of select ductal hyperplasia that show the lack of YFP in ductal hyperplasia observed in the HFD fed control mice. g′ is amplified views of select rare ducts that show VMC morphology in the Sox9-Pten mice fed HFD diet. Blue, DAPI to mark nuclei. Control mice are: *Pten *^*loxP/loxP*^*; Cre*^*-*^ mice with no treatment; *Pten*^+*/*+^*; Sox9-CreERT* mice treated with either corn oil (CO) or tamoxifen; or *Pten*^*loxP/loxP*^*; Sox9-CreERT* mice treated with coil oil.

**Figure 3:**
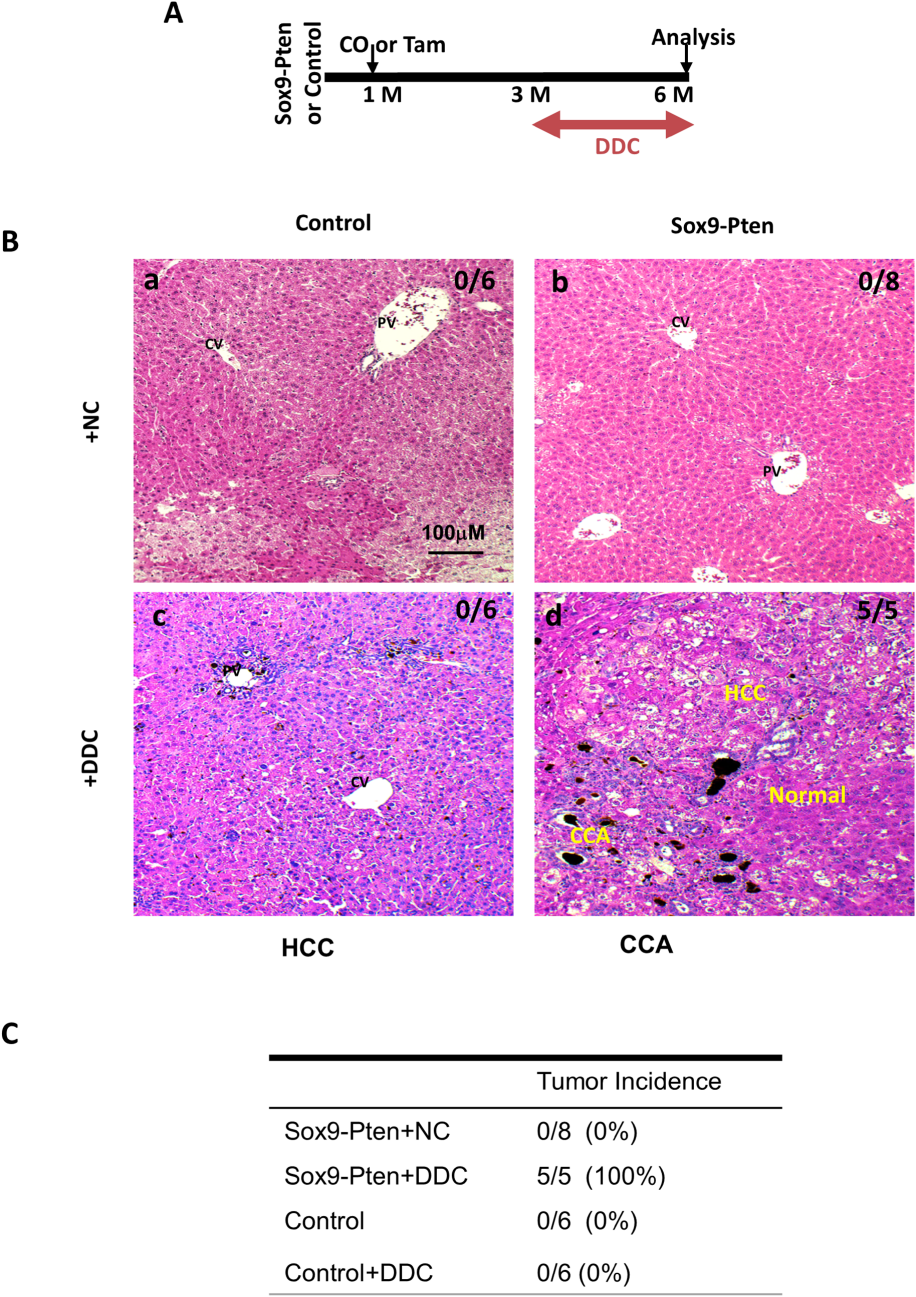

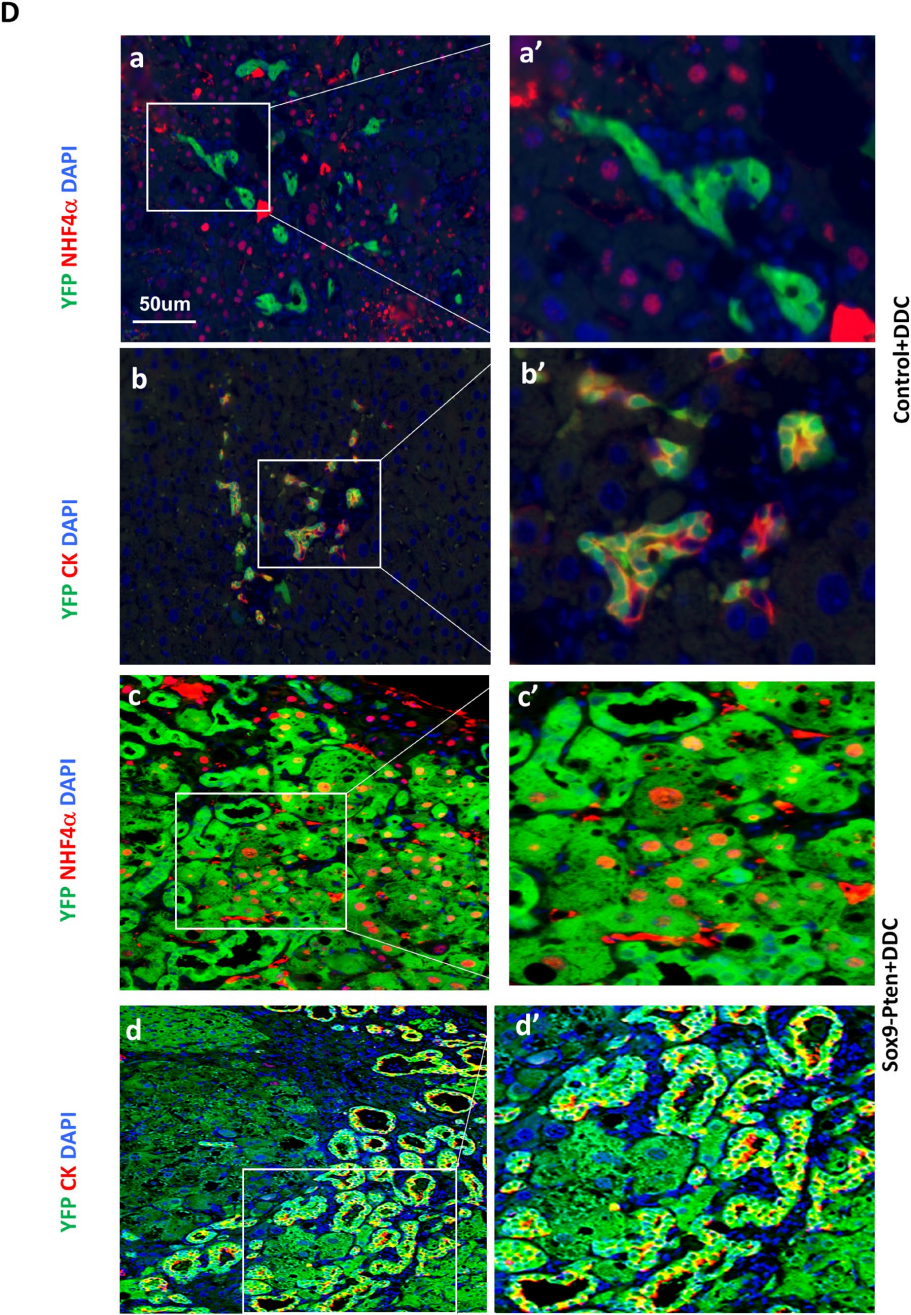
Inducing liver injury with DDC accelerates liver carcinogenesis in Sox9-Pten mice. (**A**) Protocol for tamoxifen injection and DDC treatment. Tamoxifen injected Sox9-Pten (n = 5) and control (n = 6) mice were fed on DDC for 3 months to induce liver injury before liver tissues were collected for analysis at 6 months old. Normal chow (NC) was used as diet control for DDC treatment in Sox9-Pten (n = 8) and control (n = 6) mice, respectively. (**B**) Representative microscopic images of the livers from Sox9-Pten and control mice on DDC and NC. a, Pten genotype control mice fed on NC. b. Sox9-Pten mice fed on NC. c. Pten genotype control mic fed on DDC diet. d. Sox9-Pten mice fed on DDC diet. (**C**) Quantitative analysis of tumors development in Sox9-Pten mice and control mice treated with DDC vs. NC. (**D**) Immunochemical and H&E staining on serial sections of the tumors developed in DDC treated Sox9-Pten mice vs. controls. Panels a&b, Control mice treated with DDC. Panels c & d, Sox9-Pten mice treated with DDC. Green, YFP. Red, either HNF4α (a and a′; c and c′) or CK (b and b′; d and d′). Both ductal hyperplasia in control (b and b′) and tumor formation in Sox9-Pten mice (d and d′) are positive for YFP, indicating that these are SOX9^+^ cells in origin. Panels a′-d′, amplified views of the boxed areas in a-d respectively. Blue, DAPI. Control mice are: *Pten *^*loxP/loxP*^*; Cre*^*-*^ mice with no treatment; *Pten*^+*/*+^*; Sox9-CreERT* mice treated with either corn oil (CO) or tamoxifen; or *Pten*^*loxP/loxP*^*; Sox9-CreERT* mice treated with coil oil.

**Table 1:** Phenotype of the Sox9-Pten mice.

	6 M (n = 8)^a^	8.5–9.5 M (n = 7)^a^	11–13 M (n = 22)^a^
Ductal reaction/oval cell activation^b^	7/8 (87.5%)	6/7 (85.7%)	21/22 (95.5%)
VMC^2^	5/8 (62.5%)	4/7 (57.1%)	18/22 (81.8%)
HCC^2^	0/8 (0%)	0/7 (0%)	11/22 (50%)
CCA/BDA^b^	0/8 (0%)	0/7 (0%)	14/22 (63.6%)
Heterogenous tumors (HCC + CCA/BDA)^b^	0 (0%)	0 (0%)	11/22 (55%)
BDA only^b^	0 (0%)	0 (0%)	3/22 (13.6%)
Tumors^b^	0 (0%)	0 (0%)	14/22 (63.6%)

*VMC* von Meyenburg complex, *HCC* hepatocellular carcinoma, *CCA* intrahepatic cholangiocarcinoma, *BDA* bile duct adenoma.

^a^The number of Sox9-Pten mice in each cohort at different ages;

^b^Out of the total number of mice in each cohort (the number shown after slash), the number of mice developing these phenotypes is shown before slash. The percentage of mice developing these phenotypes in each cohort is shown accordingly in parentheses.

Morphological analysis of the tumors developed in the 11–13 months old Sox9-Pten livers revealed that the tumors developed are heterogeneous, consisting of both HCC (50%, 11 out of 22, supplemental Fig. 2l) and CCA/bile duct adenoma (BDA) (63.6%, 14 out of 22, supplemental Fig. 2n and o). Figure 1A shows the presence of both CCA and HCC present in the liver of a 11–13 months Sox9-Pten mouse. Both CCA and HCC tumors are YFP positive. Immunofluorescent analysis supported that the heterogeneous tumors express both cytokeratin (CK) and hepatic nuclear factor 4α (HNF4α) in YFP^+^ tumor cells, indicating CCA and HCC, respectively (Fig. 1B). In addition, cells expressing both CK and HNF4a are also observed, representing a combined HCC-CCA phenotype (Fig. 1C) at the level of individual cells. The distinction between hepatocytes and cholangiocytes found in healthy tissue is lost in these mixed-lineage tumor cells. All tumor cells express YFP, indicating that they originate from the SOX9^+^ cells. Together, these data suggest that the cellular origin of these tumors are the targeted cells that express SOX9.

### High fat diet promotes liver injury and accelerates liver tumor development in Sox9-Pten mice

Compared to the previously reported Alb-Pten (*Pten*^*loxP/loxP*^; *Albumin-Cre*^+^) mice that carry PTEN loss in all hepatocytes^15,16^, the incidence of tumor formation in Sox9-Pten mice was significantly lower (Table 2). At 8.5–9.5 months of age, 46.7% (14 out of 30) Alb-Pten mice developed tumors whereas none of the Sox9-Pten mice developed tumors. At 11–13 months when all of the Alb-Pten mice (48 out of 48) developed tumors, the tumor incidence of Sox9-Pten mice was only 63.6% (14 out of 22). The delayed tumor onset and low tumor incidence in the Sox9-Pten mice suggest that additional signals that promote tumorigenesis are present in the Alb-Pten livers but are absent in the Sox9-Pten livers.

**Table 2:** Comparison of the Sox9-Pten with Alb-Pten mice.

	Sox9-Cre		Albumin-Cre	
8.5–9.5 M	Control^a^ (n = 7)^b^	Sox9-Pten (n = 7)^b^	Control (n = 16)^b^	Alb-Pten (n = 30)^b^
Fatty liver^c^	0/6	Periductal area	NO	Entire liver
VMC^d^	0/6	4/7 (57.1%)	0/16 (0%)	30/30 (100%)
Tumors^d^	0/6	0/7 (0%)^e^	0/16 (0%)	14/30 (46.7%)

11-13 M	Control (n = 13)^2^	Sox9-Pten (n = 22)^2^	Control (n = 126)^2^	Alb-Pten (n = 48)^2^
Fatty liver^c^	NO	Periductal area	NO	Entire liver
VMC^d^	0/13 (0%)	18/22 (81.8%)	0/126 (0%)	48/48 (100%)
Tumors^d^	0/13 (0%)	14/22 (63.6%)	0/126 (0%)	48/48 (100%)

*VMC* von Meyenburg complex.

^a^Control mice are: Pten ^loxP/loxP^; Cre^-^ mice with no treatment; Pten + / + ; Sox9-CreERT mice treated with either corn oil or tamoxifen; or Pten^loxP/loxP^; Sox9-CreERT mice treated with coil oil.

^b^The number of mice in each cohort at different ages.

^c^The location where lipids accumulate in the liver.

^d^Out of the total number of mice in each cohort (the number shown after slash), the number of mice developing these phenotypes is shown before slash. The percentage of mice developing these phenotypes in each cohort is shown accordingly in prentices.

^e^Statistically significantly different (*P* < 0.05) from that of Alb-Pten group using Fischer’s exact test.

A major difference between the phenotypes observed with Sox9-Pten and Alb-Pten livers is the penetration of steatosis^18,19^. Alb-Pten mice developed severe steatosis throughout the entire liver^15,16,19^, whereas Sox9-Pten mice only displayed mild lipid accumulation in the focal areas surrounding the tumors (supplemental Fig. 4). Sox9-Pten mice also had later tumor onset (11–13 months) as compared to Alb-Pten (8–9 months). In fact, total liver triglyceride (TG) content is actually lower in the Sox9-Pten mice compared with the control mice (supplemental Fig. 4C). Thus, we tested whether inducing steatosis would promote liver tumorigenesis in the Sox9-Pten mice by feeding the mice HFD (Fig. 2A). As expected, HFD feeding increased liver TG content and plasma alanine aminotransferase (ALT) in both control and Sox9-Pten mice (supplemental Fig. 4C&D). At nine months of age, no Sox9-Pten mice fed on NC (0 out of 7) developed tumors, but HFD feeding led to 83.3% of Sox9-Pten mice (5 out of 6) developing tumors (Fig. 2A, right panel). Feeding of HFD alone does not induce tumors in the control mice. Only mild ductal cell hyperplasia is observed in addition to lipid buildup. Interestingly, the hyperplastic ductal cells in control mice do not appear to be the SOX9^+^ ductal cells as they do not express YFP (Fig. 2B f and f′). The origin of these cells is unclear. In *Pten* deleted Sox9-Pten mice, YFP expression is observed in both ducts and periductal cells (Fig. 2B g and g′). As previously stated, VMC is observed at ducts in some mice at this age (Fig. 2B g′). HFD feeding in the Sox9-Pten mice results in tumors that are composed of YFP cells with or without CK expression (Fig. 2B d, h and h′). The CK negative cells are presumably derived from the periductal hepatocytes as they express YFP, though they adopted a pseudo-ductular structure. Overall, these results indicate that steatosis sends hyperplastic signals to SOX9^+^ cholangiocytes and periportal hepatocytes. When these cells carry oncogenic signals such as deletion of *Pten*, they are promoted to form combined HCC and CCA. Consistent with this role of steatosis, the size of tumors formed in HFD fed Sox9-Pten mice positively correlated with the magnitude of steatosis present in the liver (Supplemental Fig. 5). Out of the six Sox9-Pten mice on HFD, only one did not develop liver cancer. This mouse also had limited lipid accumulation in its liver (Supplemental Fig. 5).

**Figure 4:**
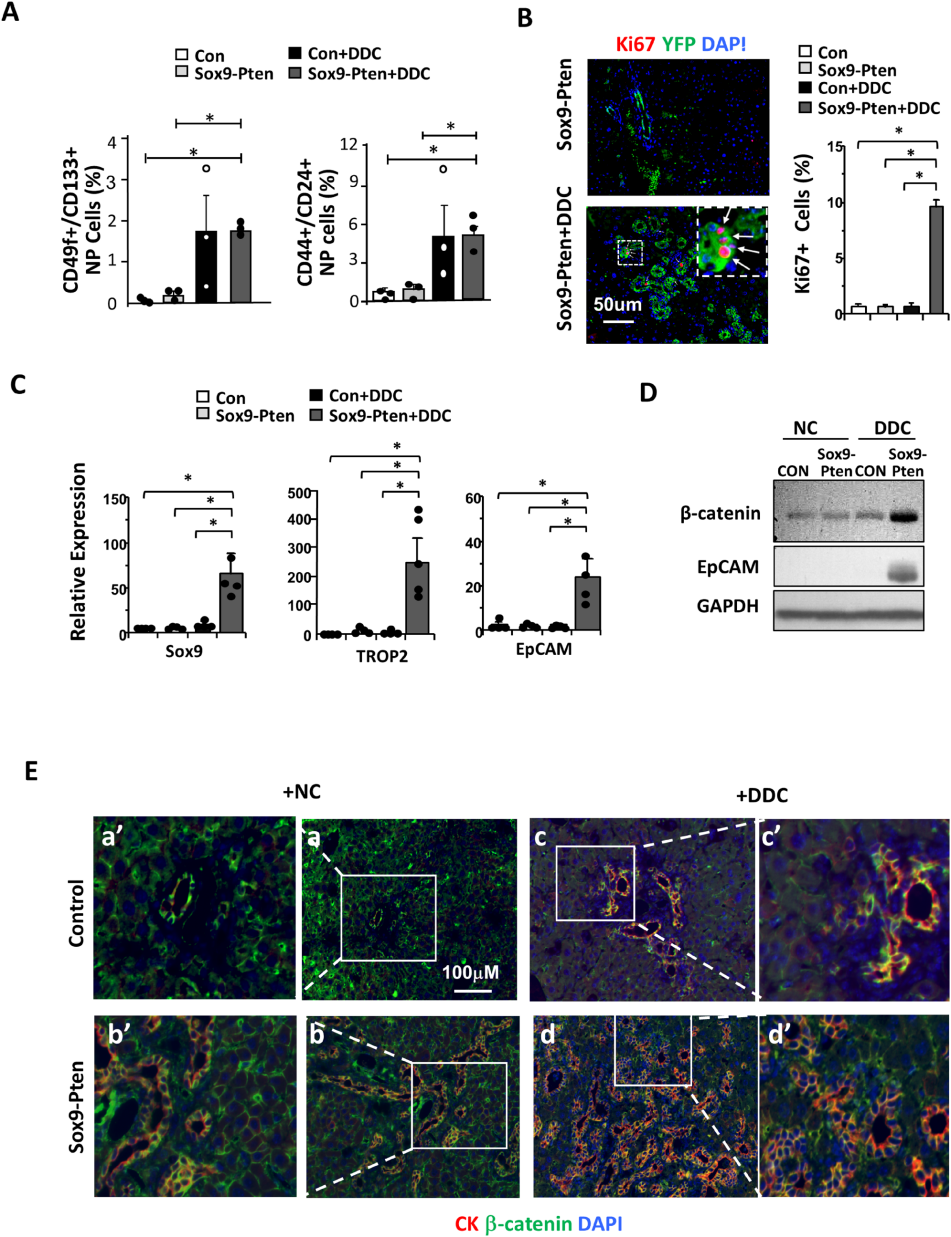
Liver injury induces the growth of tumor-initiating cells (TICs) in Sox9-Pten mice concurrent with the activation of Wnt signaling. (**A**) Accumulation of TIC populations in Sox9-Pten and control mice on DDC vs. NC (n = 3 for each group). TICs characterized by CD133 & CD49f. dual positive (left) or CD44 & CD24 dual positive (right) were significantly increased by DDC in Sox9-Pten mice. The asterisk suggests a significant difference between the two groups at *P* < 0.05. Liver cells in the control and Sox9-Pten mice treated with DDC or NC were stained for the respective cell surface CD markers followed by quantification using flowcytometer. Data expressed as percentage of dual positive cells over total nonparenchymal cells. Parenchymal cells were excluded during cell isolation. (**B**) Ki67 staining of liver sections from Sox9-Pten mice treated with DDC or NC. Left, representative image of Ki67 staining (green). Right, quantitation of Ki67 positive cells. The asterisk indicates statistical significance between the indicated groups at *P* < 0.05. Blue, DAPI. (**C**) mRNA expression of stem cell markers in control and Sox9-Pten mice treated fed DDC or NC (n = 3–5). Left, Sox9, middle, TROPS2, and right EpCAM. The asterisk indicates statistical significance between the indicated groups at *P* < 0.05. (**D**) Immunoblotting analysis of β-catenin and EpCAM in Sox9-Pten and control mice fed on DDC vs. NC. Experiment repeated at least three times with multiple individual mice. (**E**) Immunofluorescent staining of β-catenin (green) in control and Sox9-Pten livers treated with DDC vs. NC. CK (red) indicate ductal structures. Blue, DAPI.

**Figure 5:**
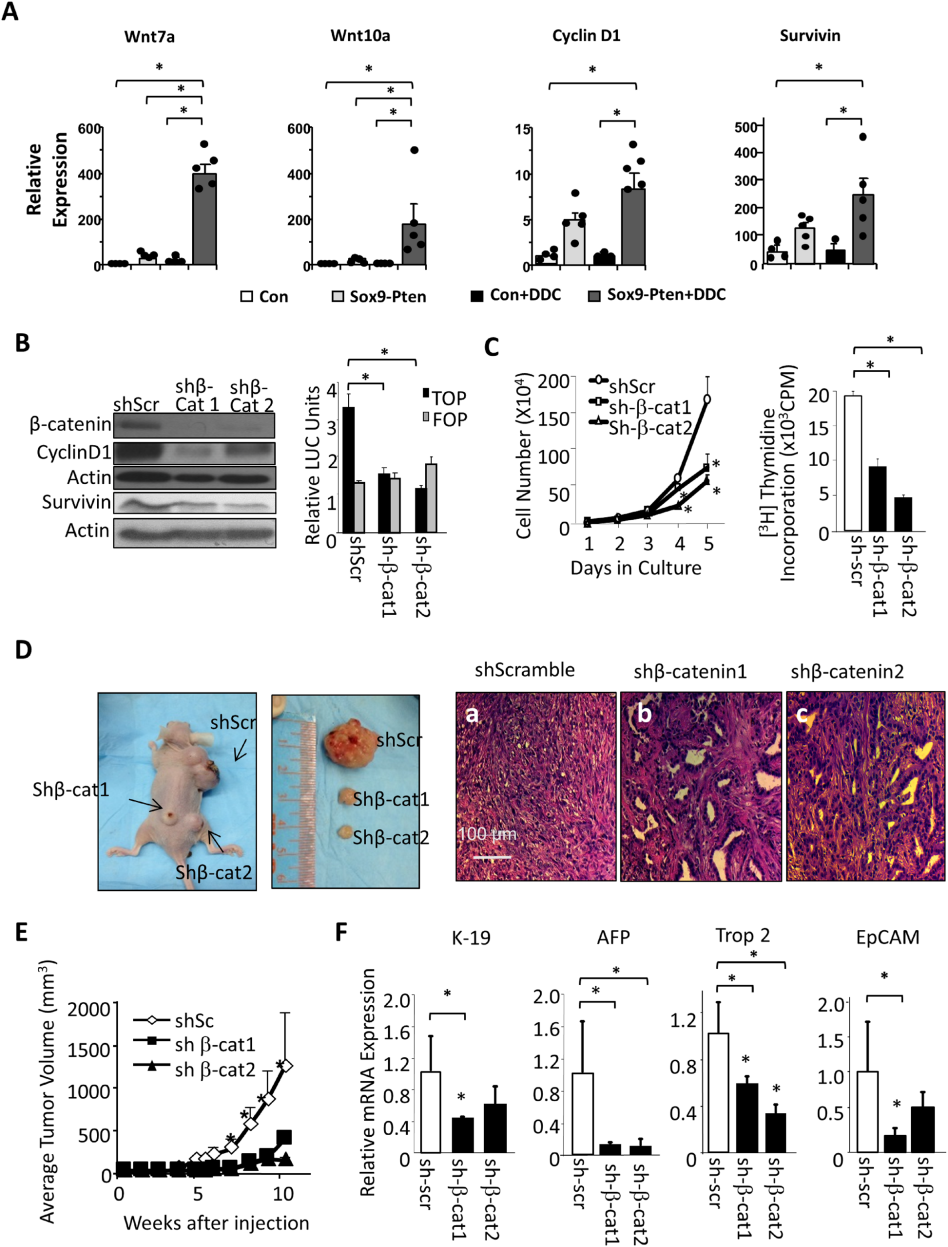
Downregulating Wnt/β-catenin attenuates TIC proliferation and reduces hepatic TIC markers. (**A**) mRNA expression of Wnt ligands (Wnt7a, Wnt10a) and downstream targets (Cyclin D1, Survivin) in Sox9-Pten mice treated with DDC vs. NC (n = 3–5). The asterisk indicates significant differences between the indicated groups at *P* < 0.05. (**B**) Confirmation of β-catenin knockdown with shRNA (shβ-Cat 1 and shβ-Cat2) vs. controls expressing sh scrambled (shScr). Left, immunoblotting of β-catenin and two transcriptional targets, cyclin D1 and survivin. Right, luciferase assay showing downregulation of transcriptional activity using a β-catenin reporter TOP (TCF reporter that contains 7 TCF response element). FOP (mutated TOP) is used as controls. n = 3. Experiments repeated at least three times. The asterisk indicates significant differences between the indicated groups at *P* < 0.05. (**C**) Growth curve (left) and thymidine incorporation (right) in β-catenin shRNA expressing cells vs. controls (shScr, shScreambled). n = 3. Experiments repeated at least three times. The asterisk indicates significant differences between the indicated groups or from the shScr group (for growth curve) at *P* < 0.05. (**D**) Macroscopic(Left two panels) and microscopic images (Right three panels, H&E) of xenographed tumors expressing shRNA for β-catenin (shβ-Cat 1 and shβ-Cat2) or controls (shScr). (**E**) Significant reduction in tumor volume observed upon β-catenin downregulation vs. scScr controls. n = 6. The asterisk indicates significant differences from both of the shβ-Cat groups at *P* < 0.05. (**F**) qPCR analysis of hepatic progenitor cell markers in xenografted tumors. n = 3. The asterisk indicates statistical significance between the indicated groups at *P* < 0.05.

### Inducing liver injury promotes tumor development in the Sox9-Pten mice

The liver steatosis in the Alb-Pten mice resulted in hepatic cell death and severe liver injury^16,20^. Our previous work and others demonstrated that suppressing liver injury effectively rescues tumorigenesis in the Alb-Pten livers^15,16,19^, suggesting that liver injury sends necessary tumor promoting signals to induce liver tumorigenesis. When the Sox9-Pten mice were fed HFD, elevated plasma ALT was observed, indicating that HFD is indeed inducing liver injury in these mice (supplemental Fig. 4D). To address whether inducing liver injury promotes the proliferation of the SOX9^+^ cells that lack PTEN and promote tumorigenesis, we fed 3-month-old Sox9-Pten mice with a diet containing 3,5-diethoxycarbonyl-1,4-dihydrocollidine (DDC) for 3 months to induce liver injury (Fig. 3A & supplemental Fig. 6). Consistent with the effect of DDC as a liver toxin^21^, DDC treatment induced an approximately fivefold increase of plasma ALT in both genotype groups at six months of age (supplemental Fig. 6B). Only mild ductal reaction and no tumors were observed in the DDC treated genotype control mice (0%, 0 out of 6). Tumor development was only observed in Sox9-Pten mice fed on DDC diet (100%, 5 out of 5) whereas none of the Sox9-Pten mice fed on NC developed any tumors at this age (0%, 0 out of 8) (Fig. 3B and C). Unlike the HFD treated mice, DDC induced ductal hyperplasia are composed primarily of YFP expressing SOX9 + cells (Fig. 3D b and b′). Immunostaining for YFP indicates tumors that originated from the targeted SOX9^+^ cells as they express YFP (Fig. 3D, c, c′, d, and d′). Dual staining for YFP with HNF4α or CK showed tumors that are positive for HNF4α and YFP as well as CK and YFP, indicating that tumors are both HCC and CCA and they originated from the targeted SOX9^+^ cells. These results suggest that liver injury promotes mixed lineage tumor formation induced by *Pten* deletion occurring in the SOX9^+^ cells.

**Figure 6:**
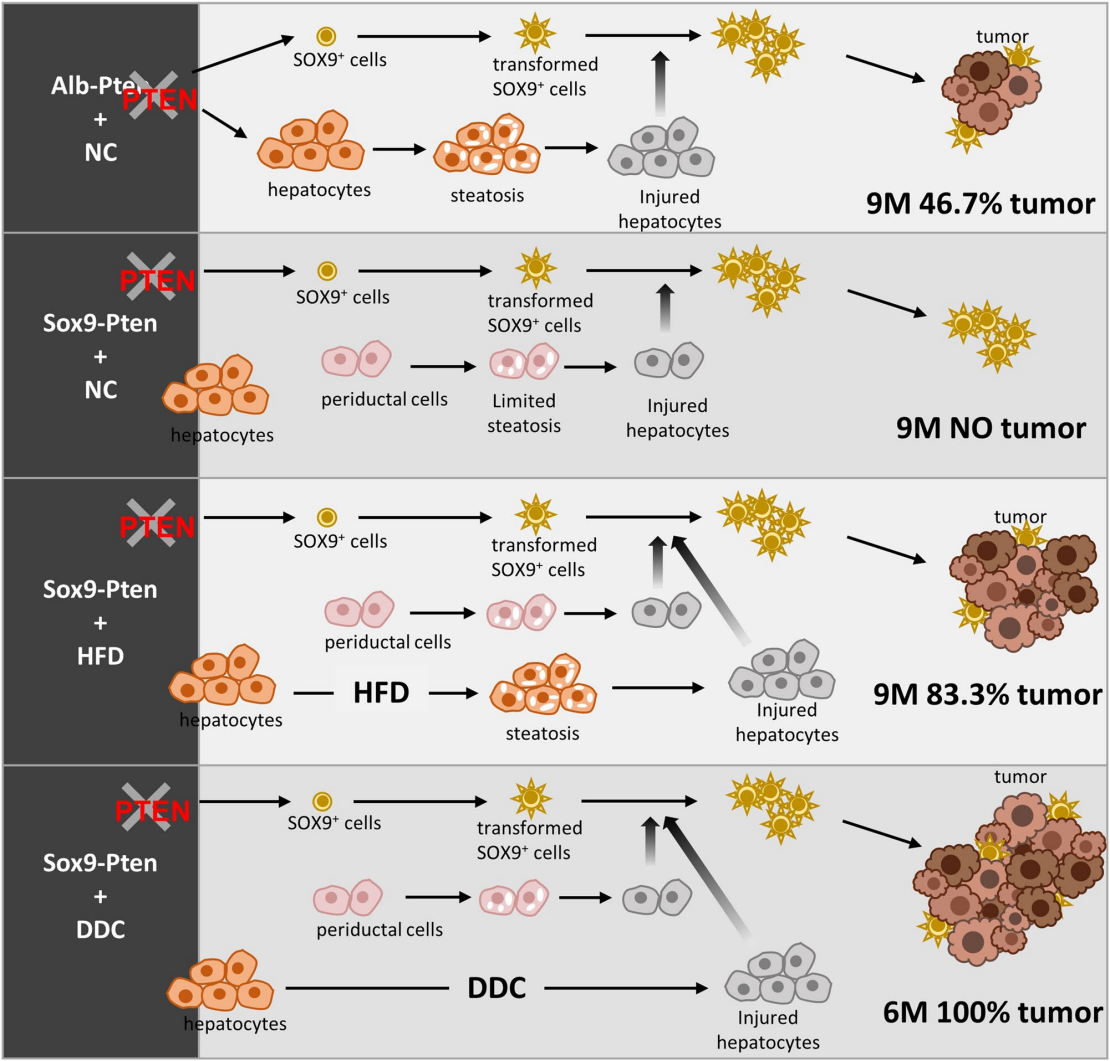
Schematic representation of the working models where liver injury promotes liver carcinogenesis from transformed SOX9^+^ cells. Using Alb-Pten and Sox9-Pten model systems, we demonstrate that both *Pten* deletion and liver injury are necessary for SOX9^+^ cells to develop into liver tumors. Because Sox9-Pten mice had less steatosis-induced liver injury compared to Alb-Pten mice, the incidence of tumor formation in SOX9-Pten mice was also lower than that of Alb-Pten mice (63.6% in Sox9-Pten vs 100% in Alb-Pten at 11–13 months old). However, with further injury induction in Sox9-Pten mice, liver carcinogenesis was accelerated in them, reaching 100% tumor incidence by DDC feeding at 6 months old and 83.3% tumor incidence by HFD feeding at 9 months old, respectively.

### Liver injury synergizes with *Pten* deletion to induce expansion of transformed SOX9^+^ cells

It has been proposed that tumor initiating cells (TICs) are the likely cellular origin of HCC and CCA^22^. A number of markers have been used in previous studies to define these TICs, including CD24, 44, 49f. and 133^23,24^. We analyzed the changes of these TIC populations in the control and Sox9-Pten mice treated with DDC for 1 month to induce TICs. As reported, feeding of DDC containing diet is capable of inducing the proliferation of TICs in the liver regardless of PTEN status (Fig. 4A and supplemental Fig. 7). Deletion of *Pten* in Sox9-Pten mice without DDC, though, does not alter the TIC populations measured with either CD24&CD44 or CD49f.&CD133 (Fig. 4A). Furthermore, very few Ki67 and YFP positive cells were found with tamoxifen injection (*Pten* deletion) alone when DDC is not present to induce liver injury (Fig. 4B). Initially *Pten* is only deleted in SOX9^+^ cells which are a small percentage of liver cells and a small portion of these are Ki67 positive indicating proliferation. Injury appears to be needed to jumpstart proliferation of *Pten* null SOX9^+^ cells. We quantified the expression of three previously defined markers for liver TICs in the liver and found that expression of SOX9, Trop 2 and EpCam are increased only in the Sox9-Pten mice treated with DDC (Fig. 4C). These observations are consistent with the morphological observation that PTEN loss alone is not sufficient to induce tumors in Sox9-Pten mice until liver injury (when steatosis occurs with aging, HFD or DDC treatment) is present.

To dissect the signals regulated by PTEN and DDC, we determined the levels of β-catenin in the liver of treated and untreated control and Sox9-Pten mice (Fig. 4D). Activating mutation of β-catenin is a key genetic event associated with liver cancer development^25^. We found here that levels of β-catenin are moderately elevated in DDC treated control livers. This moderate increase is likely due to hyperplasia of cholangiocytes that express β-catenin (Fig. 4E). PTEN loss alone has a very moderate or no effect on the expression of β-catenin. When the Sox9-Pten mice are treated with DDC, robust induction of β-catenin is observed concurrent with the expression of EpCAM (epithelial cell adhesion molecule), a β-catenin transcriptional target (Fig. 4D). Immunostaining indicates that β-catenin appears to be more pronounced in the membrane of hepatocytes in the Sox9-Pten livers vs. controls with or without DDC treatment (Fig. 4E). More cholangiocytes that coexpress β-catenin and CK are observed when either control or Sox9-Pten mice were treated with DDC, though this phenotype is much more robust in the Sox9-Pten mice. Thus, it appears that PTEN loss induces the membrane localization of β-catenin in hepatocytes but does not appear to have a significant effect on cholangiocytes until injury conditions induced with DDC are present.

We and others reported previously that expression of Wnt ligands are induced to promote liver cancer development when inflammatory conditions are present in the Alb-Pten mice^15,26^. We show here that Wnt7a and Wnt10a are only induced when both PTEN loss and injury conditions are present in the DDC treated Sox9-Pten mice (Fig. 5A). The expression of cyclin D1 and Survivin, the two targets of Wnt/β-catenin are also not significantly induced with PTEN loss alone. The moderate inductions we do see are likely a result of AKT upregulation that leads to stabilization and nuclear localization of β-catenin as previously characterized^27,28^. The expressions of these two β-catenin targets are further induced and becomes significantly upregulated with both PTEN loss and DDC treatment (Fig. 5A). Thus, both upregulation of Wnt ligand due to the combined effect of PTEN loss and tumor environmental signals and upregulation of β-catenin by PTEN controlled AKT signaling likely contributes to the induced expression of these two β-catenin target genes.

To address the effect of Wnt/β-catenin signal induction on tumor cell growth induced by PTEN loss, we introduced shRNA targeted at β-catenin to the *Pten* null tumor cell lines that we previously established^24^. In these cells, introduction of shβ-catenin led to reduced β-catenin transcriptional activity as indicated by lower TOP/FLASH reporter activity and downregulation of its target genes Cyclin D1 and Survivin (Fig. 5B and supplemental Fig. 8A & B). Downregulation of β-catenin resulted in 60% reduction in cell numbers at day 5 of culturing (Fig. 5C). A similar reduction of approximately 50% was observed for thymidine incorporation, indicating slower cell proliferation. We grafted these cells onto nude mice and observed significantly smaller tumors when β-catenin was downregulated (Fig. 5D&E and supplemental Fig. 8C). Tumor growth started to increase dramatically 4 weeks after grafting with the scramble RNA transfected cells as controls but remained barely measurable in the two β-catenin shRNA transfected samples (Fig. 5E). When the largest tumor reached the institutional allowable size, the average tumor weight in the scrambled shRNA transfected samples was 4 to 5 times the size of that in the samples transfected with β-catenin shRNA (Fig. 5E). Histological analysis shows that the small tumors carrying shβ-catenin are composed of primarily fully differentiated mature cholangiocytes (Fig. 5D). Histological appearance of the nodules resembles VMC rather than HCC or CCA. Together, these data supported a role of Wnt/β-catenin as niche factors that maintain TIC identity and growth. In both cell culture and xenografted tumors, inhibition of β-catenin led to downregulation of progenitor cell markers K-19, AFP, Trop2 and EpCAM (Fig. 5F and supplemental Fig. 8C), consistent with the role of β-catenin in maintaining TIC identity. Together, these data support a role of Wnt/β-catenin as a downstream signal that promotes the synergistic effect of PTEN loss and liver injury on liver cancer development.

## Discussion

Tumor heterogeneity has been widely observed in human liver cancer, indicative of the presence of hepatic TICs^29^. However, it is not clear what cells can serve as TICs for liver cancer. In this study, we explored the tumor initiating ability of SOX9^+^ cells given the high negative correlation between SOX9 expression and liver cancer survival. By inducing targeted deletion of tumor suppressor *Pten* in the SOX9^+^ adult liver cells, we report here that SOX9^+^ liver cells have the potential to function as liver TICs and fuel the formation of mixed-lineage tumors. In addition, we show that steatosis, a common chronic liver condition promotes the formation of tumors from these SOX9^+^ cells. We report that liver injury, which occurs with chronic steatosis, induces the proliferation of the *Pten* deletion transformed SOX9^+^ liver TICs and promotes liver cancer development. Finally, we demonstrate that Wnt/β-catenin serves as the synergistic signal that promotes the proliferation of and tumor formation from the PTEN loss transformed SOX9^+^ liver TICs.

In tumor samples from liver cancer patients, high SOX9 expression is associated with advanced tumor stage, higher tumor grade, poorer recurrence-free survival, and poorer overall survival^8,9^. Using flow cytometry to isolate SOX9^+^ cells from human HCC cell lines, the isolated cells are also positive for TIC markers, such as EpCAM and CD133 among others^10^. These isolated SOX9^+^ cells are able to divide both symmetrically to maintain self-renewal and asymmetrically to produce SOX9^-^ progenies, and fulfill various aspects of TICs including resistance to chemotherapy and capacity to initiate tumor formation in vivo^8,10^. Therefore, SOX9 has been recognized as an important liver TIC maker. The ability of SOX9^+^ cells to contribute to liver cancer formation in vivo however had heretofore not been established. At the same time, SOX9 is also considered a marker for differentiation that marks the fully differentiated mature cholangiocytes^30^. Our results here validate these works in vitro and provide in vivo evidence that SOX9^+^ cells can serve as TICs to promote tumorigenesis in the liver. When a genotoxic event, i.e. *Pten* deletion, is targeted to the SOX9^+^ cells, they are capable of forming heterogeneous tumors. This study demonstrates the nature of SOX9^+^ cells as the cellular origin for liver cancer and implicates their roles in liver cancer heterogeneity.

The SOX family of transcriptional factors play diverse roles in development and organogenesis. SOX9 plays an essential role in the embryonic development of many tissues and organs including the liver^6^. In the developing liver, expression of SOX9 is first detected in the portal side of primitive ductal structures and later on the entire bile ducts as the biliary structures mature^31^, and gives rise to both cholangiocytes and hepatocytes. In adult liver, SOX9 expression is restricted to the cholangiocytes lining the bile ducts. Previous studies that label the SOX9^+^ cells with lineage tracers have suggested that SOX9 expression marks the adult liver progenitor cells^6,7,32^, while other studies with similar approaches but labeling different populations of cells led to conflicting conclusions^33–35^. A recent study discovered low SOX9-expressing hepatocytes surrounding the portal vein and offered some explanation for this discrepancy^7^. However, that work is inconclusive for the contribution of the SOX9^+^ cells to liver carcinogenesis due to the low-level expression of detoxification genes required to metabolize carcinogens used to induce tumors in the study. Our study, by specifically targeting *Pten* deletion to SOX9^+^ cells provides strong evidence that these cells are capable of serving as TICs in the liver and likely contribute to the heterogeneity observed in liver cancer.

Liver TICs share features with normal liver progenitor cells^29^, such as self-renewal and differentiation, and express similar markers to those identified for liver progenitor cells, such as EpCAM^36^, CD 133^5,24,37^, CD44^36^, CD24^38^, Keratin19^39^, Lgr5^40^ and more recently SOX9^10^. Using these markers characterized for liver TICs, cells sorted from human HCC cell lines (e.g., Huh7, PLC/PRF/5 etc.) are also clonogenic and capable of forming spheres and xenograft tumors containing heterogeneous progenies^5,36,38–40^, a hallmark of TICs-derived tumors. However, because these studies showing tumor-initiating activity are based on sorted cells using flow cytometry, such observations cannot be validated using biopsied patient sample specimens due to insufficiently small number of cells. Our study here shows that the SOX9 expressing YFP positive cells are enriched with cells expressing markers for TICs, indicative that the SOX9 expression marks a subpopulation of the TICs established in the literature.

In experimental transgenic mouse models, previous studies from our lab and others showed that PTEN loss in the liver (using Alb-Cre) led to a primary fatty liver condition that resulted in cancer formation^16,19,32,41^. While additional insults and mutations can further promote the development of tumors^15,16,42–46^, the presence of steatosis and resulting cell death indicate that different cell types within the liver may respond differently to this genotoxic event. In the present study, the delayed tumor burden and onset in the Sox9-Pten compared to the Alb-Pten mice suggest that PTEN loss in SOX9^+^ cells alone can drive the biliary hyperplasia phenotype but additional signals are needed for tumor promotion (Fig. 6). This is consistent with clinical observations where liver cancer often develops within the framework of chronic liver diseases where sustained inflammation and tissue repair predispose the injured livers to malignancies over time^47,48^. As a common chronic disease, fatty liver disease is recognized as a crucial factor linked to increased risk of liver cancer^47,48^. Previously, using Alb-Pten mouse model combined with a caloric restriction approach or deletion of *Akt2*, the liver AKT isoform predominantly regulating metabolism in vivo, our group established that lipid accumulation that develops in the Alb-Pten is indispensable for liver carcinogenesis^15,16,19,20^. Using the Sox9-Pten mice, our study here shows that the genotoxic event occurring in the putative SOX9^+^ TICs is propagated by steatosis induced by HFD feeding (Fig. 6). Consistent with the notion that chronic fatty liver disease leads to hepatotoxicity and establishes the environment for tumor growth^49^, fibrosis is observed in the Sox9-Pten mice accompanying tumor development. Furthermore, DDC treatment that induces liver injury also promotes tumor formation in the Sox9-Pten mice (Fig. 6).

Previously, we reported that steatotic liver injury signals the production of Wnt by infiltrating macrophages and thus establishes a niche in favor of the growth of hepatic TICs^15^. Here, we demonstrate that Wnt serves as the signal to promote tumorigenesis from the SOX9^+^ TICs. Activating mutations of the primary Wnt signal effector β-catenin (*CTNNB1)* is one of the most frequent events (15.9–32.8%) identified in liver cancer by genome-wide studies^25,50^. Our results here show that the Wnt/β-catenin signal is needed to sustain proliferation, survival and self-renewal of the PTEN loss transformed SOX9^+^ liver TICs. PTEN loss and liver injury combined are needed to induce Wnt signaling and promote the advanced tumor formation phenotype observed with HFD or DDC treated Sox9-Pten mice. SOX9 has been shown to positively regulate the Wnt/β-catenin signal. Overexpressing SOX9 in naturally SOX9^-^ cells (e.g. hepatocytes) results in increased accumulation of β-catenin in the nucleus and enhanced gene expression of Wnt pathway targets, whereas SOX9 knockdown in SOX9^+^ cells causes the opposite results^10^. This effect of SOX9 on Wnt/β-catenin signaling is likely mediated by the upregulation of Frizzled-7, a key receptor of the canonical Wnt pathway that is transcriptionally regulated by SOX9^9^. Thus, in addition to marking the TIC population, induction of SOX9 resulting from PTEN loss in combination with liver injury likely contributes to the stabilization of β-catenin in these cells. This stabilization of β-catenin is necessary for the TICs to contribute to tumor formation as HCC development has been shown to result from tumor progenitors that retain β-catenin activity^33^.

In summary, we demonstrate the tumorigenicity of SOX9^+^ cells by targeted deletion of tumor suppressor *Pten.* We report here that SOX9^+^ cells have the potential of giving rise to mixed-lineage tumors. To initiate liver carcinogenesis, SOX9^+^ cells require 1) oncogenic transformation (e.g., PTEN loss-of-function) to confer them with TIC features and a growth advantage, and 2) the presence of liver injury in combination with oncogenic signals (e.g. PTEN loss-of-function) that induces microenvironmental niche (e.g., Wnt) to stimulate the proliferation of transformation-primed SOX9^+^ cells for TIC activation. While moderate steatotic injury in the periductal area of aged Sox9-Pten mice is sufficient to activate transformed SOX9^+^ cells over time, liver carcinogenesis is accelerated when liver injury is exacerbated by hepatotoxin DDC or HFD treatment (Fig. 6). Together, these data provide evidence that SOX9^+^ cells have the potential to be the TICs following a primary transformation event and that liver injury is necessary for promoting the activation of transformed SOX9^+^ cells, which eventually give rise to liver tumors with mixed lineages.

## Materials and methods

### Ethical approval

All animals were housed in a temperature, humidity and light-controlled room (12 h light/dark cycle), allowing free access to food and water. All experimental procedures were approved by the Institutional Animal Care and Use Committee (IACUC) guidelines at the University of Southern California. All methods were performed in accordance with the relevant guidelines and regulations. The study was carried out in compliance with the ARRIVE guidelines.

### Animals

*Pten*^*loxP/loxP*^; *Albumin-Cre*^+^ (Alb-Pten) mice have been previously characterized^18^. *Pten*^*loxP/loxP*^; *Sox9-Cre*^*ERT*+^;* R26R*^*YFP*^ (Sox9-Pten) mice were generated through breeding *Pten*^*loxP/loxP*^ mice with *Sox9-Cre*^*ERT*+^*; R26R*^*YFP*^ mice^23^. Cre activity in Sox9-Pten mice was induced by subcutaneous injection of three doses of tamoxifen (125 mg/kg, Sigma-Aldrich, St Louis, MO, USA) every other day at 1 month of age. Control mice are: Pten ^loxP/loxP^; Cre^-^ mice with no treatment; Pten^+/+^; Sox9-CreERT mice treated with either corn oil (CO) or tamoxifen; or Pten^loxP/loxP^; and Sox9-CreERT mice treated with coin oil.

Both male and female mice were used for the initial tumor screening study. Subsequent diet and liver toxin intervention studies used male mice only to minimize sample size needed based on power analysis. Mice were randomly assigned to treatment vs. vehicle groups for induction of gene deletion as well as diet intervention.

### Diet feeding and drug treatment in vivo

For injury induction, a diet containing 0.05% 3,5-dietoxycarbonyl-1,4 dihydrocollidine (DDC) (Newco Distributor, Rancho Cucamonga, CA, USA) and a high fat diet (HFD)—60 kcal% fat diet (06,414 Harlan Laboratories, Indianapolis, IN, USA) were given to mice, respectively as indicated. Mice fed normal chow (NC) were used as non-injury controls.

### Cell culture, plasmids, transfection and luciferase assay

Mouse hepatic TIC cells were isolated as the CD133^+^ non-parenchymal cell population from the *Pten* deleted mice^24^. The established TICs were cultured in Dulbecco’s Modified Eagle’s Medium/F12 (Corning Inc., Corning, NY, USA) supplemented with 10% FBS (VWR International, Radnor, PA, USA), supplemented with 5 μg/ml insulin (Sigma-Aldrich, St Louis, MO, USA), 10 ng/ml epidermal growth factor (Invitrogen, Carlsbad, CA, USA), and phenobarbital (2 mM). All cells were incubated at 37 °C with 85% relative humidity and 5% CO2.

shRNAs directed against β-catenin or shScramble (control) were transfected into cells using Lipofectamin 3000 (Thermo Fisher Scientific, Waltham, MA, USA) according to the manuals. Briefly, 1.0 × 10^4^ hepatic TIC cells were seeded into two sets of 6 well plates. Cells were allowed to attach overnight; they were then transfected with shScramble (control) and shβ-catenin followed by puromycin selection. TICs were also co-transfected with shRNA β-catenin constructs and with either TOPFLASH or FOPFLASH luciferase to monitor the effect of shRNA. Cells were harvested 48 h after transfection. Transfection efficiencies were normalized using Renilla luciferase activity. Luciferase assays were performed using the Dual-Luciferase Reporter Assay System (Promega, Madison, WI, USA). Sequence of the shRNA used are provided in supplemental Table 1.

### Cell growth, thymidine incorporation and xenograft

Cell growth is evaluated by counting the number of cells in each well at 24-h intervals for five days after initial seeding of TICs transfected with shRNA directed against β-catenin or shScramble (control) in 6 well plates. Media was refreshed every two days until the conclusion of the experiment. Growth curve analysis was based on experiments done in triplicate. For thymidine incorporation analysis, TICs were incubated with 0.5 μCi/well [^3^H] thymidine for 48 h at 37 °C following puromycin selection. Cells were then washed with phosphate-buffered saline (PBS) and 0.5 ml of 10% trichloroacetic acid was added to precipitate the DNA at the conclusion of the 48-h incubation period. Unbound thymidine was then removed with PBS. 500 μl of 0.1% Triton X and 10% sodium hydroxide were used to dissolve DNA. Final lysates were transferred to scintillation vials and mixed with scintillation fluid in 1:3 ratio and counted on Perkin Elmer Luminescence Counter.

To graft tumors, TICs transfected with shScramble (control), shβ-catenin 1 and shβ-catenin 2 were then injected subcutaneously onto nude mice. Tumor volume was monitored weekly using a calibrator.

### Flow cytometry analysis

Enriched non-parenchymal progenitor cell population was obtained by mincing and digesting liver followed by CD45 depletion using Miltenyi Biotec magnetic beads as described previously^15^. After blocking, one million cells were incubated for 30 min with primary antibodies: CD133 (Miltenyi-Biotec, Bergisch Gladbach, Germany), Streptavidin eluor 450 (e-Bioscience Inc., San Diego, CA, USA), Anti-human/mouse CD49f.-APC (eBioscience Inc., San Diego, CA, USA), Anti-mouse CD133-FITC (e-Bioscience Inc., San Diego, CA, USA). Cells were then washed in PBS and analyzed using Cyan (Bechman Coulter, Brea, CA, USA) and Calibur. Further analysis was conducted using Flow-Jo program.

### Immunopathology and immunohistochemistry

Zn-formalin fixed and paraffin embedded liver sections were stained as reported^16^. Hematoxylin and eosin (H&E) staining was performed for morphology analysis. For immunohistochemistry analyses, antibodies including YFP (Abcam, Cambridge, MA, USA), HNF4α (Abcam, Cambridge, MA, USA), Cytokeratin (CK, Dako Omnis, Santa Clara, CA, USA), Ki67 (Thermo Fisher Scientific, Waltham, MA, USA), SOX9 (Millipore, Burlington, MA, USA) were used. Liver sections were also co-stained with DAPI for nuclei.

### RNA isolation, reverse transcription, and quantitative PCR

Total RNA was extracted from liver tissues using Trizol reagent (Invitrogen, Carlsbad, CA, USA) following manufacturer’s instructions. Reverse transcription was performed with M-MLV reverse transcriptase system (Promega, Madison, WI, USA) using 2 μg of total RNA. Quantitative PCR was performed using PowerUP Syber Green Master Mix (Thermo Fisher Scientific, Waltham, MA, USA) and 7900 HT Fast Real-Time PCR System (Applied Biosystem, Grand Island, NY, USA)^51^. Gene-specific primers are listed in supplemental Table 1. Relative expression of mRNA levels was determined by the delta-delta Ct method. Housekeeping gene GAPDH was used as internal control.

### Protein electrophoresis and immunoblotting

Tissues or cell lysates (75–150 ug protein) were subjected to SDS-PAGE, followed by transferring proteins from gel to polyvinylidene fluoride (PVDF) membrane (Bio-Rad Laboratories, Hercules, CA, USA) for immunoblotting. PVDF membranes were probed with antibodies against β-catenin (Cell Signaling Technology, Danvers, MA, USA), EpCAM (Abcam, Cambridge, MA, USA), and Cyclin D1 (Cell Signaling Technology, Danvers, MA, USA), Survivin (Cell Signaling Technology, Danvers, MA, USA), Actin (Sigma-Aldrich, St Louis, MO, USA), GAPDH (Thermo Fisher Scientific, Waltham, MA, USA).

### Biochemical and molecular biology assays

Hepatic lipids were extracted using Folch method by adding chloroform/methanol (2/1). The supernatant was used for triglyceride (TG) assay using TG (GPO) Reagent Set (Thermo Fisher Scientific, Waltham, MA, USA). Plasma ALT level was assessed using ALT Reagent (Raichem, San Diego, CA, USA) as previously described^16^.

### Statistical analysis

Difference between two groups were analyzed by two-tailed Student’s t tests. For multigroup comparison, a one-way analysis of variance (ANOVA) was conducted followed by post-hoc analysis using REGW Q test. A *p* value < 0.05 is considered statistically significant. Data were presented as mean ± SEM.

## Supplementary information

Supplementary Information.
