# Supplementary material for: Transformation of SOX9+ cells by Pten deletion synergizes with steatotic liver injury to drive development of hepatocellular and cholangiocarcinoma
Source: Sci Rep. 2021 Jun 3;11:11823. doi: 10.1038/s41598-021-90958-1 (PMC8175600; doi:10.1038/s41598-021-90958-1)
Supplement: Supplementary file 1 — Supplementary Information. [file 41598_2021_90958_MOESM1_ESM.pdf]

A

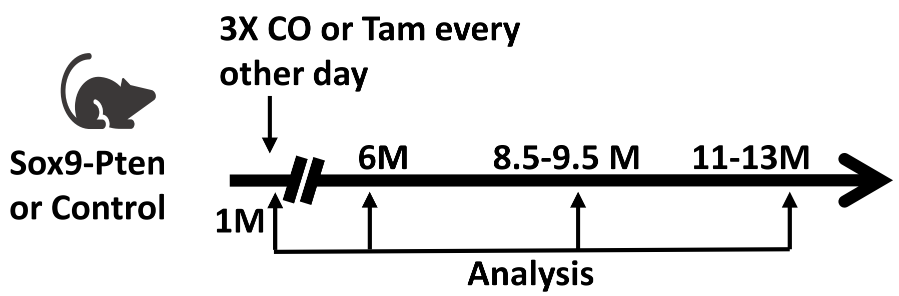

B

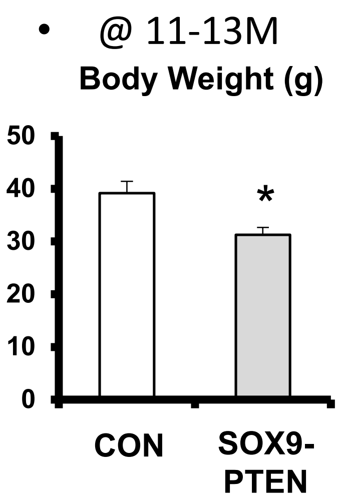

C

• 3 days post last dose of TAM injections

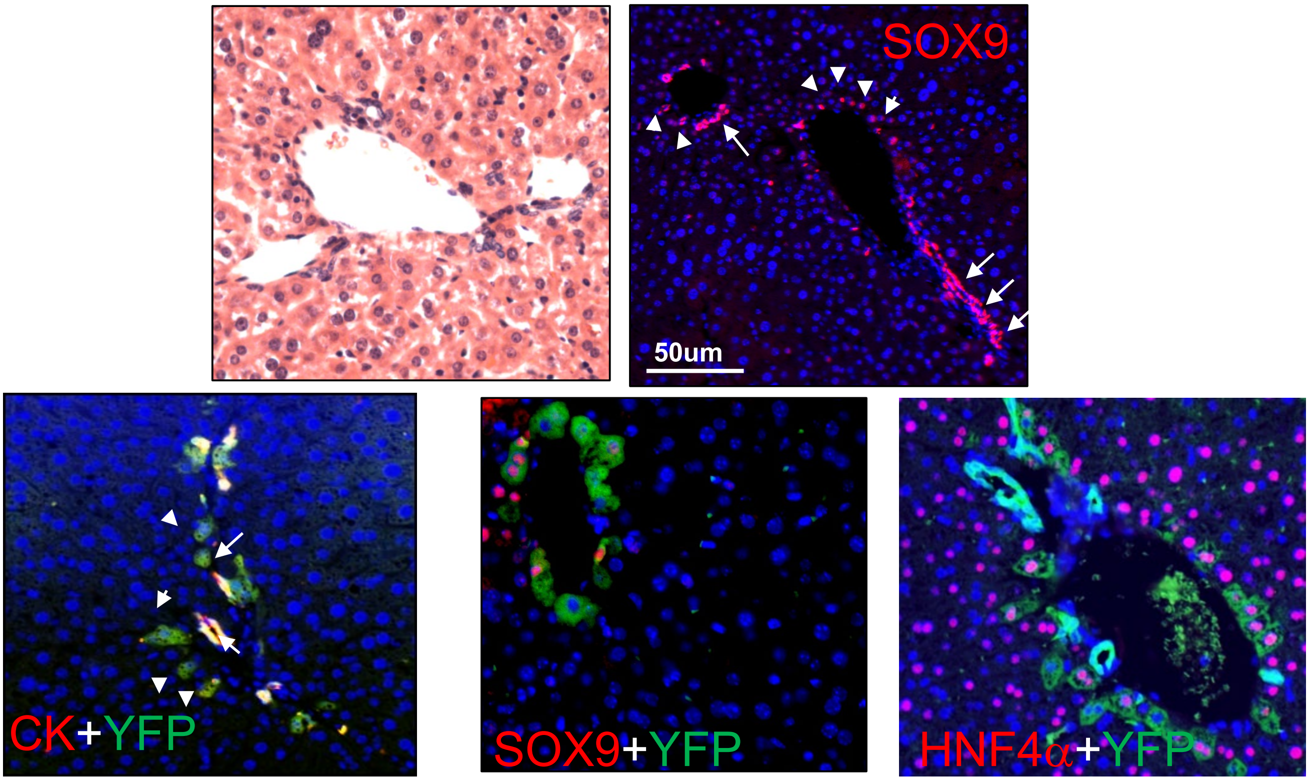

Supplemental Figure 1. Characterization of Sox9-Pten mice. (A) Tamoxifen injection and analysis protocol. Sox9-Pten and control mice received three doses of tamoxifen (Tam) every other day at 1 month old, and liver tissues were analyzed three days after the last dose of tamoxifen and at 6, 8.5-9.5, and 11-13 months old. Control mice are: Pten $^{loxP/loxP}$; Cre^{-} mice with no treatment; Pten+/+; Sox9-CreERT mice treated with either corn oil (CO) or tamoxifen; or Pten$^{loxP/loxP}$; Sox9-CreERT mice treated with coil oil. **(B)** Body weight comparison of Sox9-Pten (n=18) vs. control mice (n=13) at 11-13 months old. The asterisk suggests a significant difference from control mice at P<0.05. **(C)** Representative H&E and SOX9 (red), CK (red) + YFP (green), SOX9 (red) + YFP (green) staining on liver tissues collected from Sox9-Pten mice three days after the last injection of tamoxifen. Immunochemical staining shows that YFP was expressed by the cells in the periportal area.

Chen et al Supplemental Figure 2

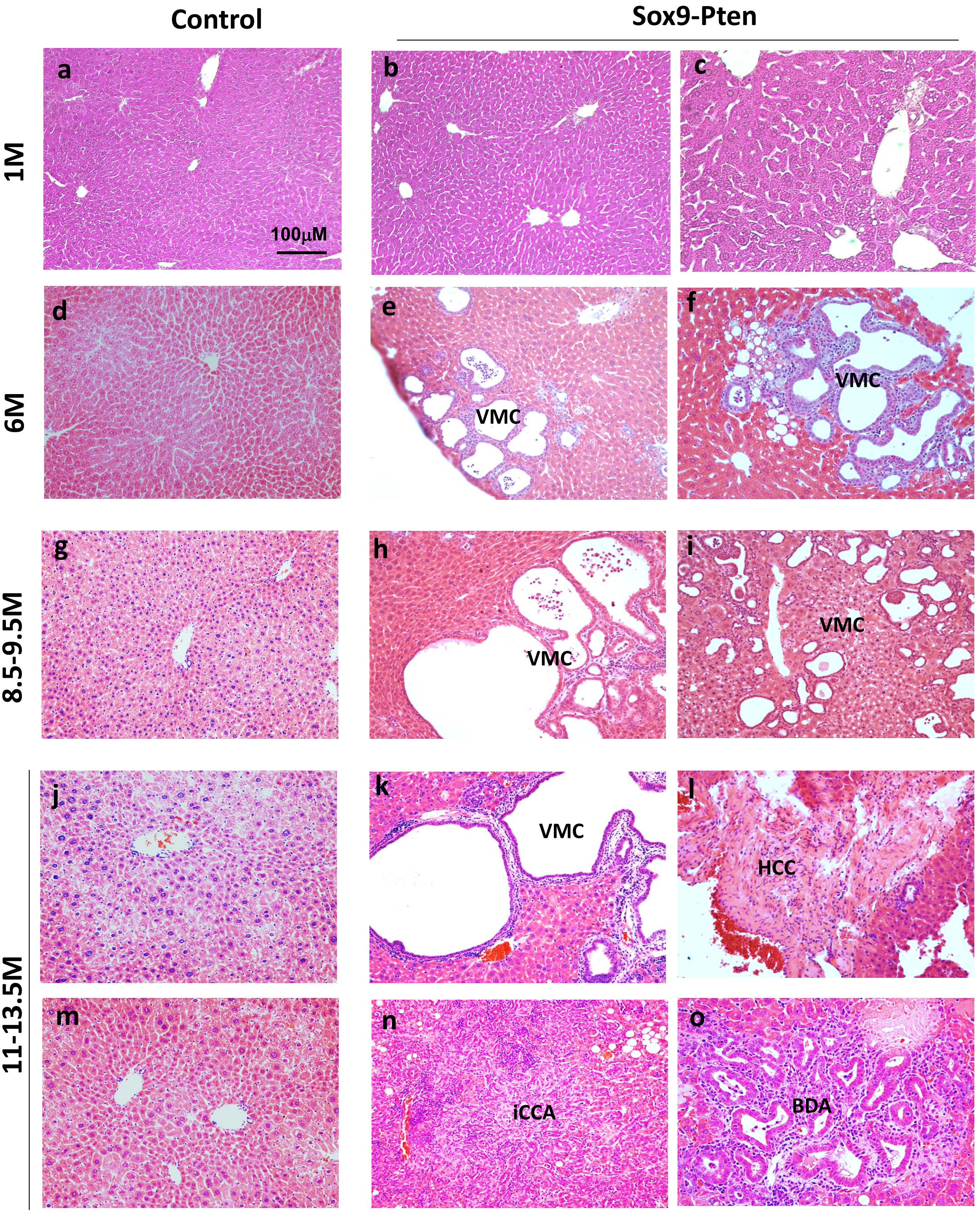

Supplemental Figure 2 Morphological analysis of Sox9-Pten vs. control mice at different ages.

H&E stained liver tissues showing abnormal morphologies observed in different ages of the Sox9-Pten and control mice, 1 month (n=3), 6 months (Sox9-Pten, n=8; Control, n=6), 8.5-9.5 months (Sox9-Pten, n=7; Control, n=3), and 11-13 months (Sox9-Pten, n=22; Control, n=18). VMC conditions are observed in focal areas of 50-60% of the mice starting at 6 months old. Tumors are observed at 11-13 months old in half of the mice. The tumors developed in Sox9-Pten mice exhibited clinical features of HCC, and iCCA/BDA.

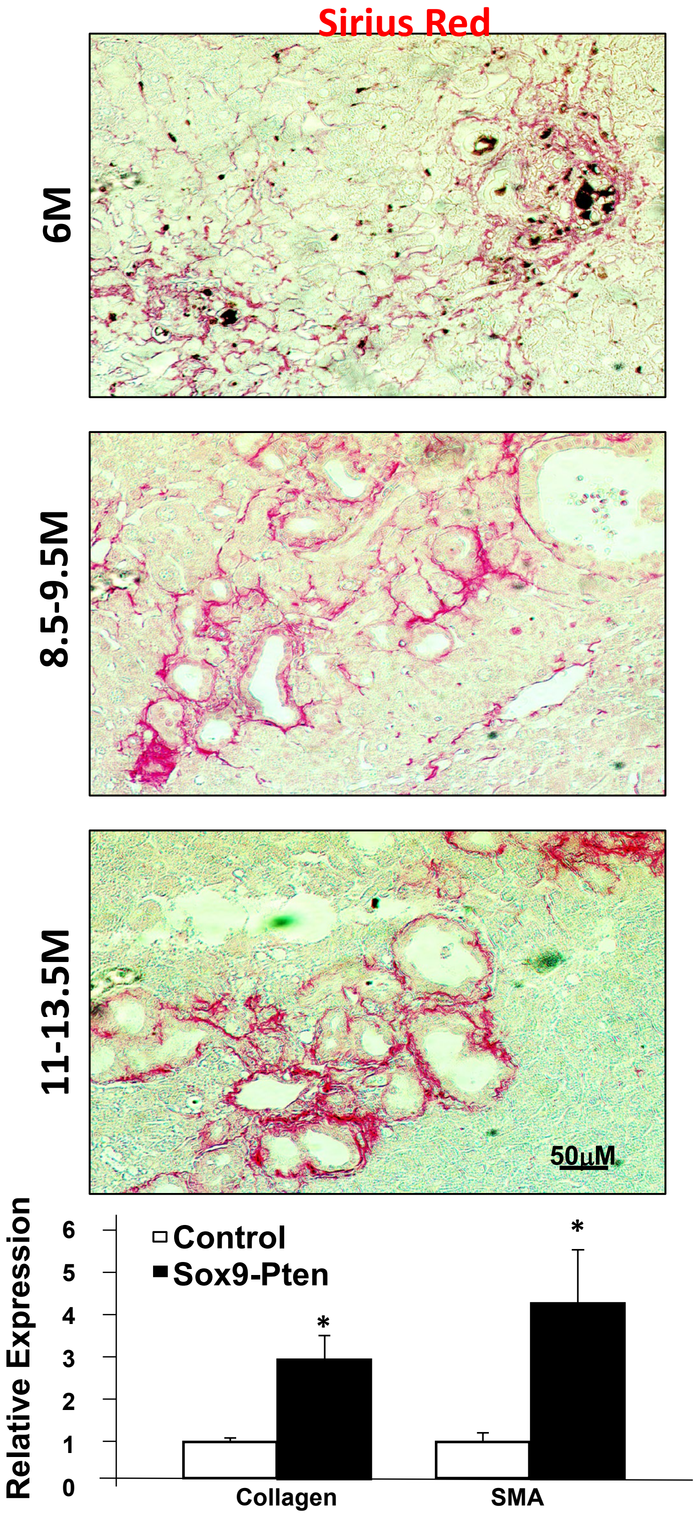

Supplemental Figure 3 Sirius Red stainging of Sox9-Pten vs. control mice at different ages.

A. Sirius Red staining is performed in 6, 9 and 12 months old Sox9-Pten mice. n=6. The staining shows progressive more intensive Sirius Red staining as the mice progress to tumor stage from 6 to 12 months of age.

B. Relative mRNA expression of collagen and Smooth muscle a actin (SMA). n=3. $p<0.05$ vs. control.

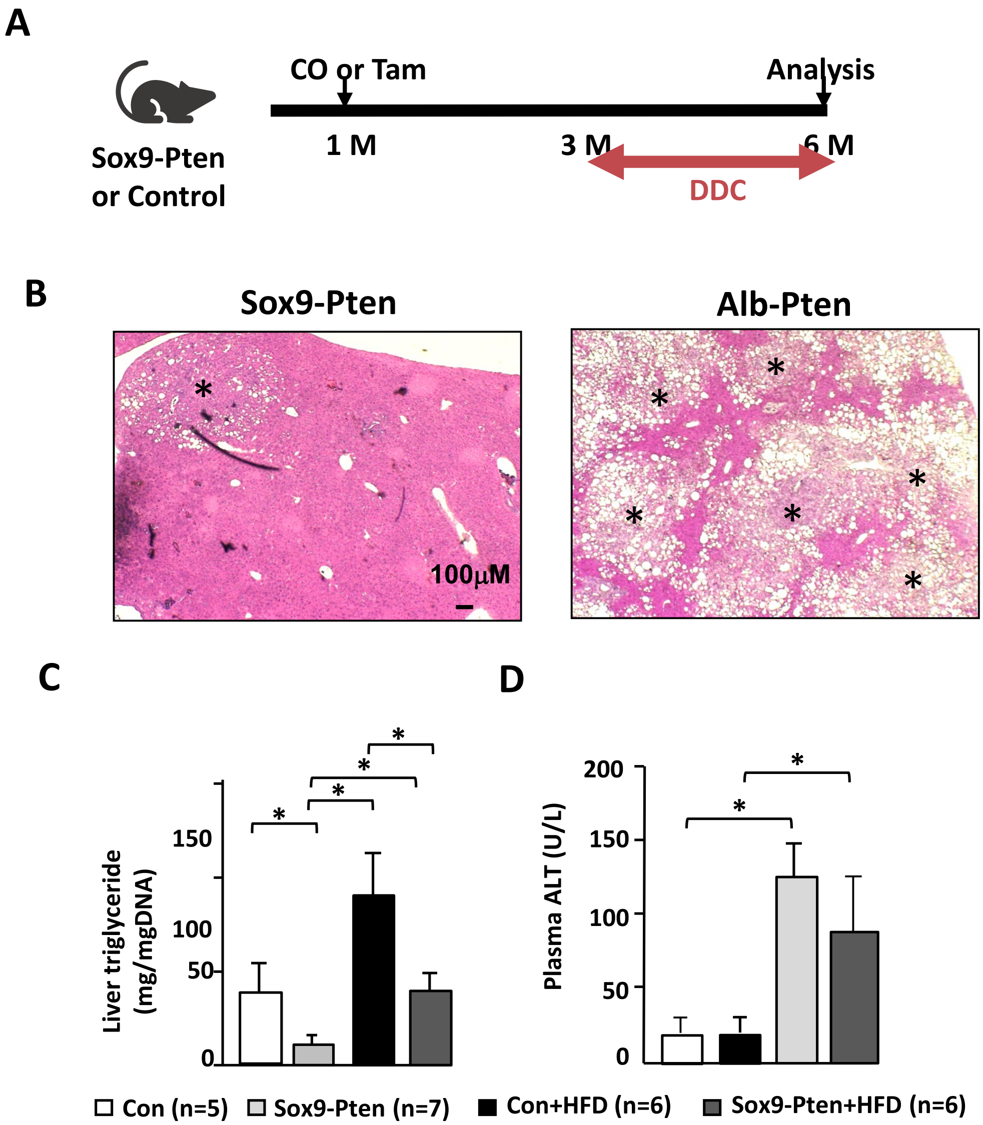

Supplemental Figure 4 Lipid droplets are accumulated in the area surrounding focal lesions in Sox9-Pten mice.

(A) Tamoxifen injection and diet protocol for the HFD experiment. **(B)** Representative H&E images of liver tumors in Sox9-Pten and Alb-Pten mice at 12 months old. Sox9-Pten mice exhibited mild steatosis in the periductal region surrounding the tumors, whereas Alb-Pten mice developed massive steatosis throughout the entire liver. The asterisk indicates the tumor area. **(C)** Liver triglyceride levels in Sox-Pten and control mice on HFD vs NC. **(D)** Plasma ALT in different groups of mice. The asterisk indicates statistical significance at $P<0.05$.

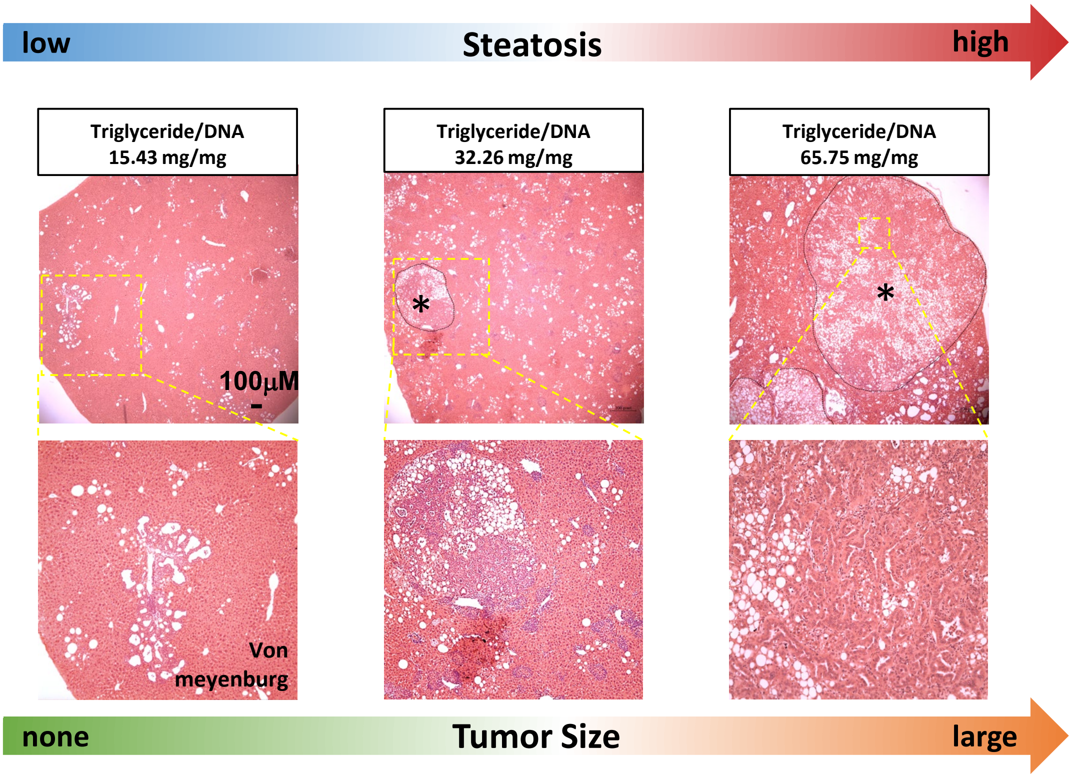

Supplemental Figure 5 High-fat diet accelerates liver carcinogenesis in Sox9-Pten mice.

Representative H&E images of the livers from Sox9-Pten mice on HFD. Liver triglyceride levels correlate with the size of liver tumors in these mice. Liver TG levels in the one mouse that only developed VMC is in the range of mice fed on NC (left).

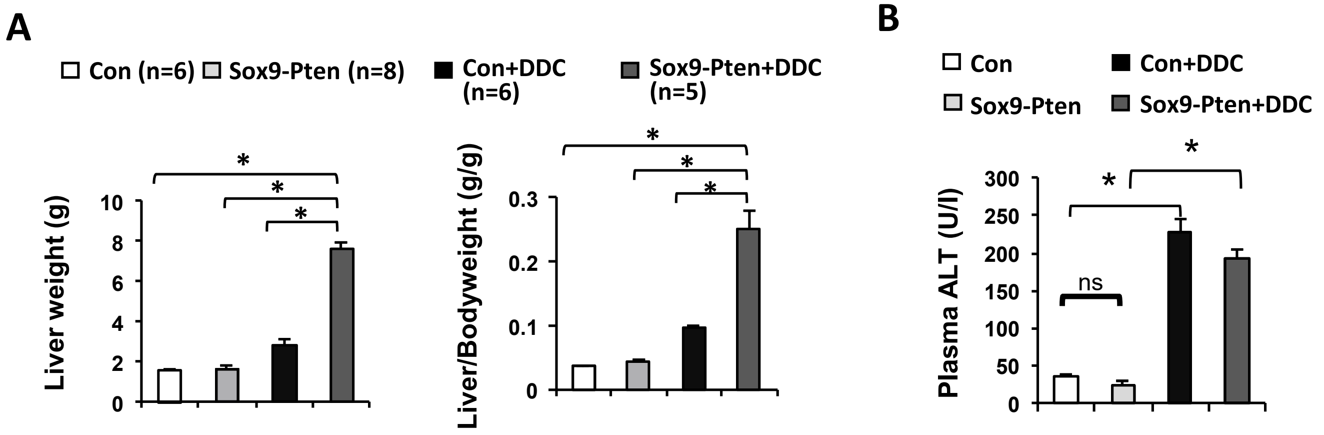

Supplemental Figure 6 DDC treatment induces liver injury in Sox9-Pten mice.

A) Liver weight was significantly increased in Sox9-Pten vs. control mice by DDC (Left), so was liver to body weight ratio (Right). The asterisk suggests a significant difference from DDC fed Sox9-Pten mice **(B)** Plasma ALT levels were elevated in DDC fed Sox9-Pten and control mice, indicating the presence of liver injury. The asterisk indicates statistical significance at P<0.05.

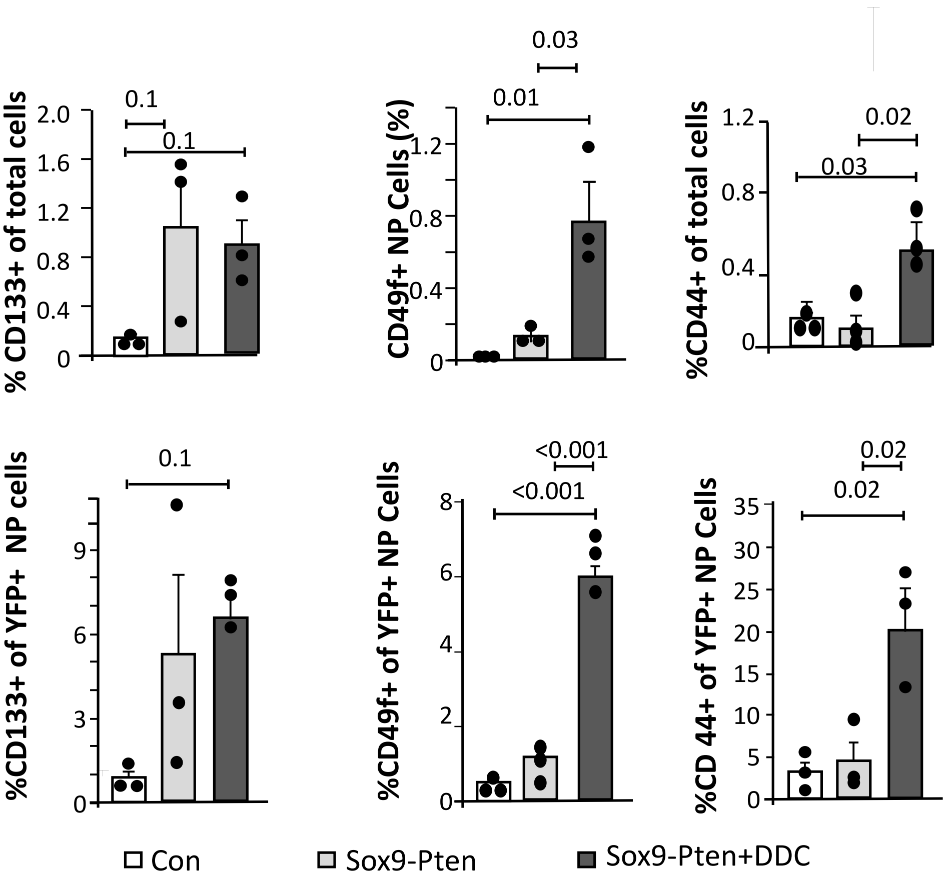

Supplemental Figure 7 Liver injury induces the growth of tumor-initiating cells (TICs) in Sox9-Pten mice in concurrent with the activation of Wnt signaling.

Analysis of TIC populations in Sox9-Pten and control mice on DDC vs. NC (n=3 for each group). TICs characterized by CD133, CD49f and CD44 were analyzed in (top) total non-parenchymal (NP) or (bottom) YFP^{+} NP cell population.

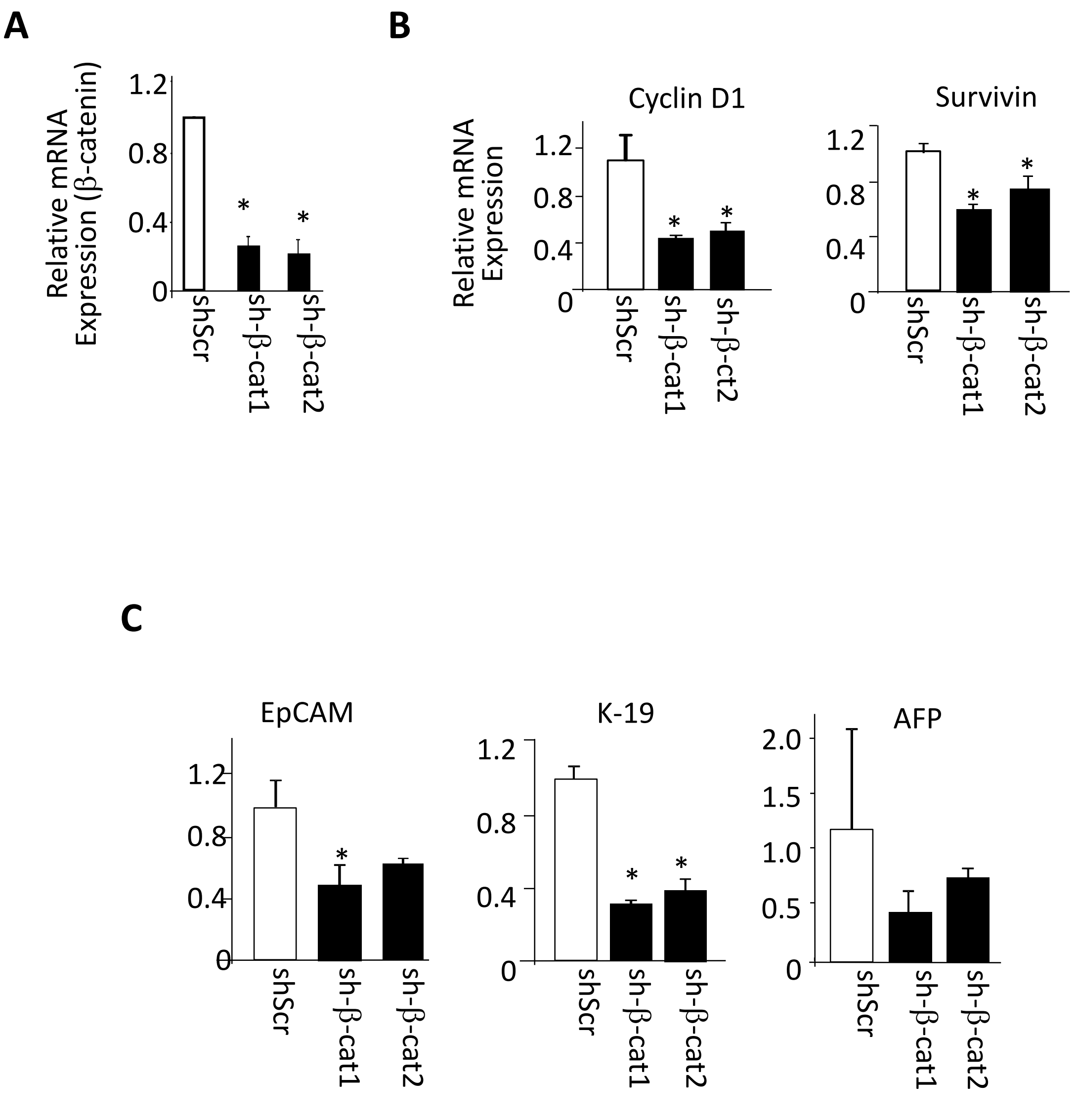

Supplemental Figure 8. Downregulating Wnt/β-catenin attenuates TIC proliferation and reduces hepatic TIC markers. Confirmation of the effect of β-catenin targeting shRNAs on the expression of β-catenin (**A**) Cyclin D1 and Survivin (**B**). qPCR analysis of hepatic progenitor cell markers *in* cultured TICs (**C**). n=3. The asterisk indicates values that are significantly different from sh-Scr group at $P<0.05$.

Supplemental table 1 Primers used for quantitative PCR and sequence for shRNA against β-catenin

Gene/shRNA	Primer Probe	Sequence
SOX9	Forward	GTGCAAGCTGGCAAAGTTGA
	Reverse	TGCTCAGTTCACCGATGTCC
EpCAM	Forward	AGGGGCGATCCAGAACAACG
	Reverse	ATGGTCGTAGGGGCTTTCTC
Trop2	Forward	AGACCAAAGCCTGCGCTGCG
	Reverse	AGCTGGGGTGCAGCTTGTAG
Wnt7a	Forward	CGACTGTGGCTGCGACAAG
	Reverse	CTTCATGTTCTCCTCCAGGATCTTC
Wnt10a	Forward	GACTCCACAACAACCGTGTG
	Reverse	CCTACTGTGCGGAACTCAGG
Cyclin D1	Forward	TCCGCAAGCATGCACAGA
	Reverse	GGTGGGTTGGAAATGAACTTCA
Survivin	Forward	ACTACCGCATCGCCACCT
	Reverse	GACGGTTAGTTCTTCCATCT
K19	Forward	CCGGACCCTCCCGAGATTA
	Reverse	CTCCACGCTCAGACGCAAG
AFP	Forward	ATCGACCTCACCGGGAAGAT
	Reverse	GAGTTCACAGGGCTTGCTTCA
GAPDH	Forward	GTCGGTGTGAACGGATTTGG
	Reverse	GACTCCACGACATACTCAGC
shβ-catenin 1	Forward	GATCCGATGTTGACACCTCCCAAGTTCAAGAGACTTGGG AGGTGTCAACATCTTTTTGGAAA
	Reverse	AGCTTTTCCAAAAAGATGTTGACACCTCCCAAGTCTCT TGAACTTGGGAGGTGTCAACATCG
shβ-catenin 2	Forward	GATCCGGCTTTCCCAGTCCTTCATTCAAGAGATGAAGGA CTGGGAAAAGCCTTTTTGGAAA
	Reverse	AGCTTTTCCAAAAAGGCTTTTCCCAGTCCTTCATCTCTT GAATGAAGGACTGGGAAAAGCCG

Uncropped Immunoblotting Images

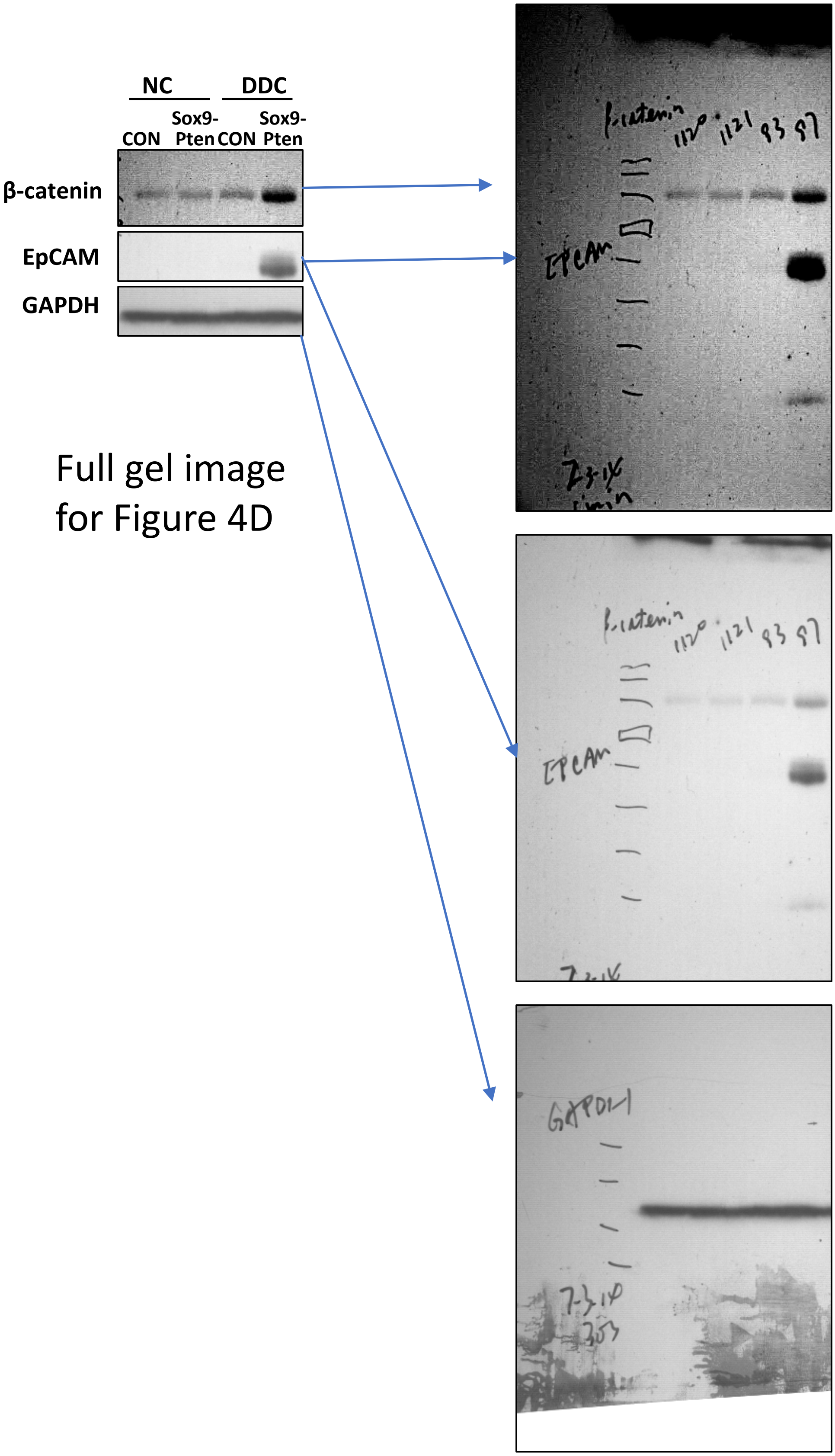

Full gel image for Figure 4D

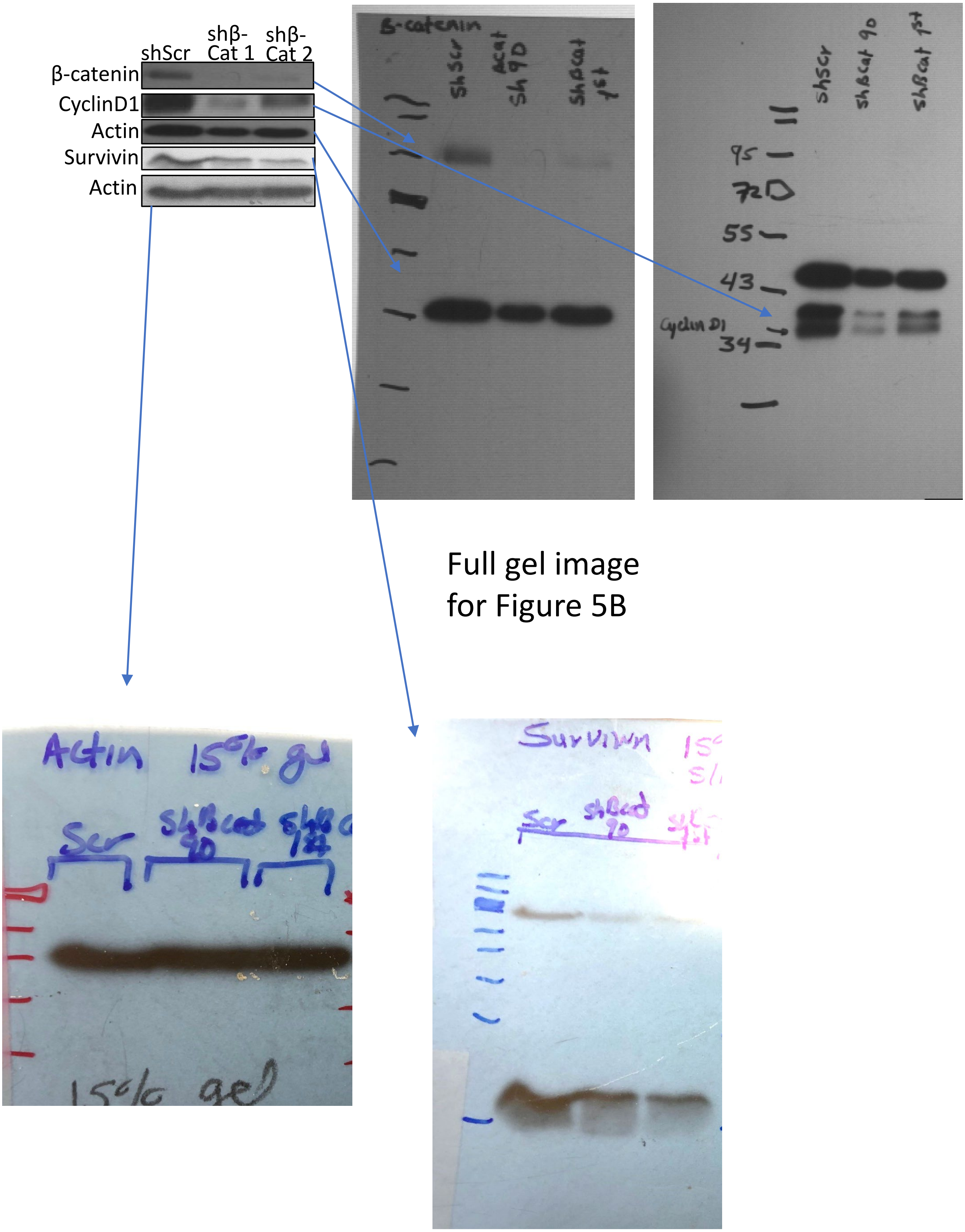

Full gel image for Figure 5B
